# CEACAM1 induces B-cell survival and is essential for protective antiviral antibody production

**DOI:** 10.1038/ncomms7217

**Published:** 2015-02-18

**Authors:** Vishal Khairnar, Vikas Duhan, Sathish Kumar Maney, Nadine Honke, Namir Shaabani, Aleksandra A. Pandyra, Marc Seifert, Vitaly Pozdeev, Haifeng C. Xu, Piyush Sharma, Fabian Baldin, Florian Marquardsen, Katja Merches, Elisabeth Lang, Carsten Kirschning, Astrid M. Westendorf, Dieter Häussinger, Florian Lang, Ulf Dittmer, Ralf Küppers, Mike Recher, Cornelia Hardt, Inka Scheffrahn, Nicole Beauchemin, Joachim R. Göthert, Bernhard B. Singer, Philipp A. Lang, Karl S. Lang

**Affiliations:** 1 Institute of Immunology, Medical Faculty, University of Duisburg-Essen, Hufelandstrasse 55, Essen 45147, Germany; 2 Clinic of Gastroenterology, Hepatology and Infectious Diseases, Heinrich-Heine-University, Moorenstrasse 5, Düsseldorf 40225, Germany; 3 Institute of Cell Biology (Cancer Research), University of Duisburg-Essen, Virchowstrasse 173, Essen 45122, Germany; 4 Clinic for Primary Immunodeficiency, Medical Outpatient Unit and Immunodeficiency Laboratory, Department of Biomedicine, University Hospital, Basel 4031, Switzerland; 5 Department of Physiology I, University of Tuebingen, Gmelinstrasse 5, Tuebingen 72076, Germany; 6 Institute of Medical Microbiology, Faculty of Medicine, University Hospital Essen, Hufelandstrasse 55, Essen 45122, Germany; 7 Institute of Virology, University of Duisburg-Essen, Hufelandstrasse 55, Essen 45147, Germany; 8 Clinic of Gastroenterology and Hepatology, University of Duisburg-Essen, Hufelandstrasse 55, Essen 45147, Germany; 9 Rosalind and Morris Goodman Cancer Centre, Departments of Biochemistry, Medicine and Oncology, McIntyre Medical Science Building, Montreal, Quebec, Canada H3G 1Y6; 10 Department of Hematology, West German Cancer Center (WTZ), University Hospital Essen, Hufelandstrasse 55, Essen 45147, Germany; 11 Institute of Anatomy, Medical Faculty, University of Duisburg-Essen, Hufelandstrasse 55, Essen 45147, Germany; 12 Department of Molecular Medicine II, Heinrich Heine University Düsseldorf, Universitätsstrasse 1, Düsseldorf 40225, Germany

## Abstract

B cells are essential for antiviral immune defence because they produce neutralizing antibodies, present antigen and maintain the lymphoid architecture. Here we show that intrinsic signalling of CEACAM1 is essential for generating efficient B-cell responses. Although CEACAM1 exerts limited influence on the proliferation of B cells, expression of CEACAM1 induces survival of proliferating B cells via the BTK/Syk/NF-κB-axis. The absence of this signalling cascade in naive *Ceacam1*^*−/−*^ mice limits the survival of B cells. During systemic infection with cytopathic vesicular stomatitis virus, *Ceacam1*^*−/−*^ mice can barely induce neutralizing antibody responses and die early after infection. We find, therefore, that CEACAM1 is a crucial regulator of B-cell survival, influencing B-cell numbers and protective antiviral antibody responses.

## Abstract (web summary)

Antibody responses are regulated by selective survival of B cells with proper antigen specificity. Here the authors show that CEACAM1 is critical for B-cell survival during homeostasis and antiviral responses.

## Graphical abstract

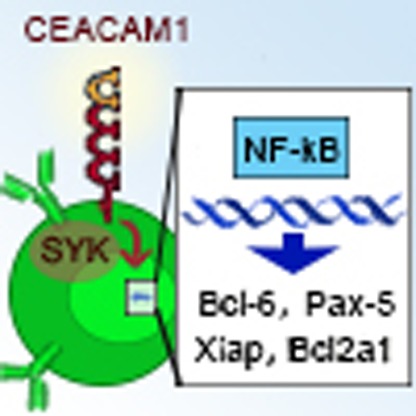

B cells are central players in initiating a rapid antiviral immune response. Their main functions are producing virus-specific antibodies, presenting antigen and participating in building the splenic architecture^1^,^2^,^3^,^4^. Three subsets of B cells are important contributors to immune responses against pathogens: B1, marginal zone (MZ) and follicular B cells^5^,^6^. B1 B cells usually seed the peritoneal and pleural cavities and develop T-cell-independent antibody responses against bacterial antigens^7^. B1 B cells are also responsible for generating the so-called natural antibodies that are detectable in naïve mice that have not experienced antigen^7^. MZ B cells are located in the splenic MZ, where they have direct contact with blood-borne pathogens. Therefore, antigen-activated MZ B cells usually respond hours after infection and build the specific antibody response early after infection^5^. Antigen-activated follicular B cells move to germinal centres, where the antibody’s affinity matures, and switch classes by recombining to mount long-lasting high-affinity immunoglobulin G (IgG) antibody responses against pathogens^5^.

Once B cells leave the bone marrow, two important signals determine their fate. First, tonic signalling by the B-cell receptor (BCR) in the absence of antigen is essential for the further differentiation and survival of mature B cells^8^. Second, signalling via the B-cell-activating factor (BAFF) receptor strongly contributes to B-cell survival^9^. BCR activation of B cells leads to phosphorylation of Bruton’s tyrosine kinase (BTK), a member of the Tec family of non-transmembrane protein tyrosine kinases (PTKs)^10^,^11^. BTK phosphorylation after BCR ligation leads to the activation of canonical nuclear factor-κ light-chain enhancer of activated B (NF-κB) cell pathway, in addition to nuclear factor of activated T (NFAT) cells and extracellular signal-regulated kinase (ERK) pathways^12^,^13^. Crosslinking of the BAFF receptor activates the NF-κB pathway non-canonically via NF-κB-inducing kinase (NIK) and inhibitor of NF-κB, IκB kinase 1 (ref. 14). Although BAFF receptor signalling was first believed to be independent of BCR signalling, a recent report suggested that BAFF receptor signalling may also include the BCR signalling pathway components^15^. The NF-κB pathway substantially contributes to B-cell survival by inducing the expression of Bcl-2, Bcl-x_L_ and Mcl-1 (ref. 13).

The carcinoembryonic antigen-related cell adhesion molecule 1 (CEACAM1), a member of the carcinoembryonic antigen and the immunoglobulin families, is engaged in intercellular binding interactions that affect various signal transduction pathways associated with cell proliferation and differentiation^16^,^17^. CEACAM1 usually acts via intercellular adhesion through homophilic (CEACAM1–CEACAM1) or heterophilic (CEACAM1–CEACAM5, CEACAM1–CEACAM6 and CEACAM1–CEACAM8) interactions^17^,^18^. In mice, there are at least four CEACAM1 isoforms: CEACAM1-4L, CEACAM1-4S, CEACAM1-2L and CEACAM1-2S. The CEACAM1 ectodomain is composed of four (CEACAM1–4) or two (CEACAM1–2) highly glycosylated Ig-like domains, which are highly flexible and participate in anti-parallel (*trans*) and parallel (*cis*) homophilic binding^19^. The isoform with the short cytoplasmic tail (CEACAM1-S) can bind calmodulin, tropomyosin, actin, annexin II and PDIP38, and is phosphorylated on Ser449 through protein kinase C^20^,^21^,^22^. The isoform with the long cytoplasmic tail (CEACAM1-L) contains two additional immunoreceptor tyrosine-based inhibitory motif-carrying segments^17^,^23^. Within these immunoreceptor tyrosine-based inhibitory motif-carrying segments, phosphorylation at two tyrosines initiates signalling through CEACAM1-mediated signal transduction. Furthermore, *trans*-homophilic CEACAM1 binding induces *cis*-dimerization, and primarily dimeric but not monomeric CEACAM1-L recruits two specific Src homology region 2 domain-containing phosphatase-type phosphatases (PTPN6 and PTPN11)^19^,^24^,^25^,^26^. Crosslinking of CEACAM1 as monomers and dimers induces binding of the SRC family of PTK members to CEACAM1, which typically causes cell activation^19^,^26^.

CEACAM1 is expressed by a broad range of cell types, including angiogenically activated endothelia/lymphendothelia, various leukocyte subpopulations, normal epithelial cells and tumour cells^16^. Although *Ceacam1*^*−/−*^ mice do not exhibit this broad CEACAM1 expression, they develop normally and, in the absence of specific challenges, show no signs of disease^27^. CEACAM1 has been described primarily as a regulator of T cells in the gut^20^,^28^,^29^,^30^. Expression of CEACAM1-L inhibits T-cell proliferation and therefore prevents inflammatory bowel disease^30^. Expression of CEACAM1-S is essential for the development of follicular T helper cell-driven IgA production by gut B cells^20^. CEACAM1 also acts as a co-stimulatory molecule for T-cell receptor and BCR signalling^31^,^32^,^33^. The role of CEACAM1 in B-cell homeostasis and in antiviral B-cell responses *in vivo* remains unknown.

We report here that CEACAM1 is expressed on blood, bone marrow, lymph node, as well as splenic MZ and follicular zone (FO) B-cell subpopulations in mice. *In vitro* CEACAM1 expression induces the survival of proliferating B cells. In line with this finding, *Ceacam1*^*−/−*^ mice carry reduced numbers of total B cells and virtually no MZ B cells. During viral infection, the absence of CEACAM1 on B cells leads to an insufficient antiviral B-cell response, and *Ceacam1*^*−/−*^ mice die early after infection with the cytopathic vesicular stomatitis virus (VSV).

## Results

### CEACAM1 is expressed on B-cell subsets

**Figure 1:**
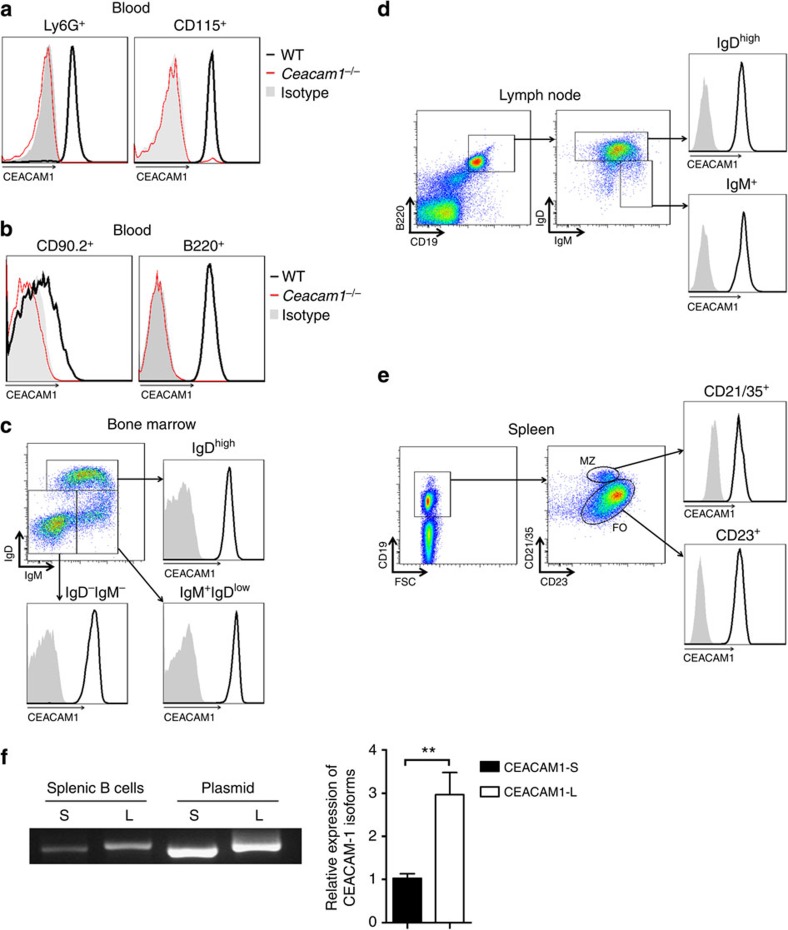
CEACAM1 is expressed on murine B-cell subsets. (**a**,**b**) Representative histogram showing CEACAM1 expression in leukocyte subpopulations isolated from wild-type (WT, black line) and *Ceacam1*^*−/−*^ mice (red line). Isotype control antibody staining of leukocytes from WT mice is shown as a grey area. Peripheral blood leukocytes gated for Ly6G (granulocytes) and CD115 (monocytes; **a**) and CD90.2^+^ (T cells) or B220^+^ (B cells) cells (**b**), as measured by flow cytometry (*n*=6 per group). (**c**–**e**) Dot plot showing IgD and IgM expression of cells gated on B220, and histogram showing CEACAM1 expression (black line) versus isotype control antibody staining (grey area) in bone marrow (**c**), lymph nodes (**d**) and spleens (**e**), respectively, from WT mice, as measured by flow cytometry (*n*=6 per group). (**f**) Representative expression levels of CEACAM1-S and CEACAM1-L isoforms in murine splenic B cells by real-time PCR (*n*=6). **P<0.01 (Student’s *t*-test). Data are representative of two (**a**–**f**) experiments (mean±s.e.m., **f**).

We first analysed CEACAM1 expression on various cell populations in the peripheral blood of wild-type (WT) mice. Erythrocytes stained negative for CEACAM1 (Supplementary Fig. 1). As previously reported^34^,^35^,^36^, high levels of CEACAM1 expression were detected on blood granulocytes (Ly6G^+^) and monocytes (CD115^+^) with the anti-CEACAM1-specific monoclonal antibody (clone CC1, Fig. 1a). Cells from *Ceacam1*^*−/−*^ mice stained negative for CEACAM1 (Fig. 1a). Next, we analysed CEACAM1 expression on lymphoid cells in the blood. CD90.2 cells, representing primarily T cells, showed weak CEACAM1 expression by individual cells (Fig. 1b), a finding suggesting that various T-cell subpopulations may differentially express CEACAM1. B cells in peripheral blood expressed CEACAM1 at high levels (Fig. 1b). Evaluation of precursor (B220^+^CD43^*−*^IgM^+^IgD^low^) and mature B cells (B220^+^CD43^*−*^IgD^high^) in the bone marrow revealed strong CEACAM1 expression in the B-cell lineage (Fig. 1c). In line with this finding, lymph node B cells, follicular B cells (FO, B220^+^CD19^+^CD21/35^lo^CD23^hi^) and MZ B cells (B220^+^CD19^+^CD21/35^hi^CD23^lo^) in the spleen expressed CEACAM1 at high levels (Fig. 1d,e). Furthermore, quantitative RT–PCR analyses of expression of long and short isoforms of CEACAM1 showed that murine splenic B cells express more long than short isoforms (Fig. 1f). In conclusion, strong CEACAM1 expression was detected in all B-cell subpopulations tested, a finding implying that CEACAM1 is functionally relevant in B cells.

### CEACAM1 induces survival genes via Syk and Erk and NF-κB

**Figure 2:**
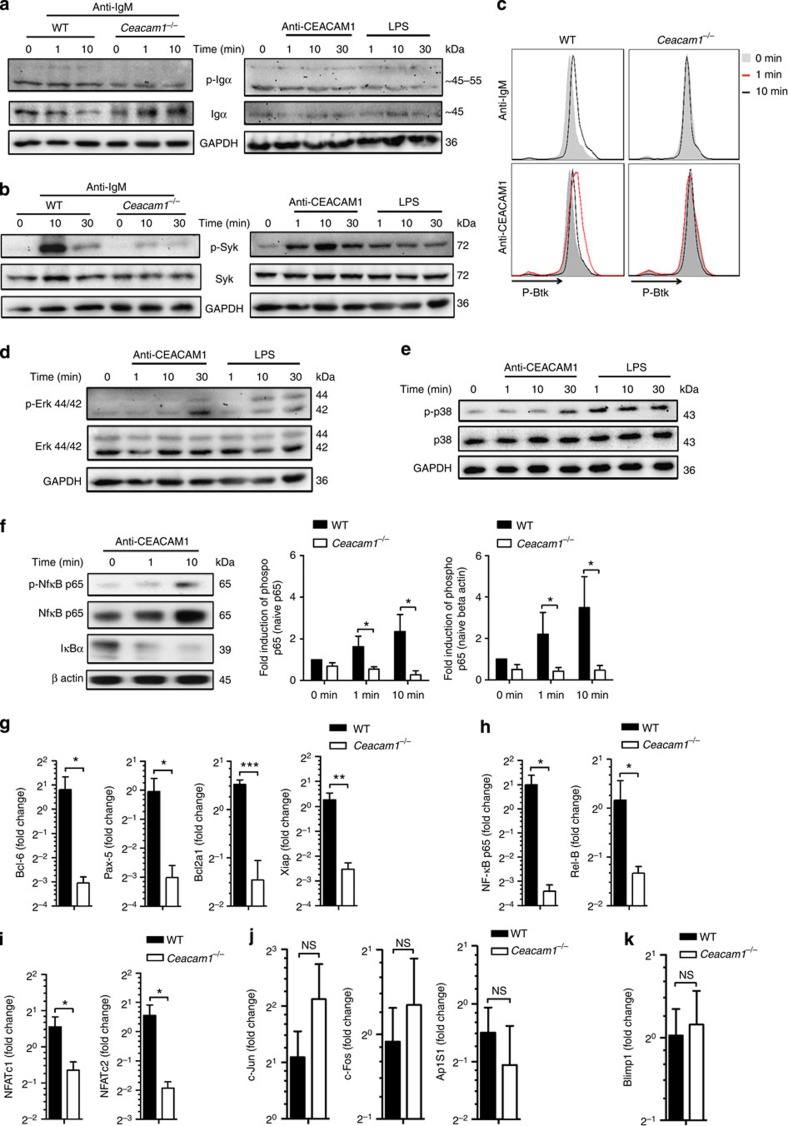
CEACAM1 in B cells induces survival genes via Syk and Erk and NF-κB. (**a**,**b**) Representative immunoblot probed with antibodies to phospho-Igα, Igα and glyceraldehyde 3-phosphate dehydrogenase (GAPDH) in wild-type (WT) and *Ceacam1*^*−/−*^ splenocytes (**a**, left panel) or in WT Splenocytes (**a**, right panel) or probed with antibodies to phospho-Syk, Syk and GAPDH in WT and *Ceacam1*^*−/−*^ splenocytes (**b**, left panel) or in WT splenocytes (**b**, right panel) after treatment with anti-IgM or anti-CEACAM1 monoclonal antibody or LPS for indicated time points (*n*=4). (**c**) Representative flow cytometry histogram of WT or *Ceacam1*^*−/−*^ splenocytes gated on B cells showing p-Btk (pY223)/Itk (pY180) staining; splenocytes were left untreated (grey area) or were treated with anti-IgM or anti-CEACAM1 antibody for indicated time points at 37 °C (*n*=3). (**d**,**e**) Representative immunoblot probed with antibodies to Tyr-P-Erk (p-p44/42 MAPK), Erk (p44/42) and GAPDH (**d**, *n*=4) or with antibodies to phospho-p38, p38 and GAPDH (**e**, *n*=3) in WT splenocytes after treatment with anti-CEACAM1 antibody or LPS for indicated time points. (**f**) Representative immunoblot and quantification of specific bands after staining for p-NF-κB p65, NF-κB p65, IκBα and β-actin in WT or *Ceacam1*^*−/−*^ B cells after challenge with anti-CEACAM1 antibody for indicated time points (*n*=4). (**g**–**k**) RT–PCR analysis of representative transcription factors such as *Bcl-6*, *Pax-5* (**g**, *n*=5), *Bcl2a1*, *Xiap* (**g**, *n*=7), *NF-κB p65, Rel-B* (**h**, *n*=5–7), *Nfatc1*, *Nfatc2* (**i**, *n*=5) or *c-Jun*, *c-Fos*, and *Ap1s1* (**j**, *n*=5) or *Blimp1* (**k**, *n*=7) mRNA from WT and *Ceacam1*^*−/−*^ B cells sorted from spleen and 24 h after without stimulation (values show fold change to expression in B cells 0 h after sorting). *P<0.05; **P<0.01 and ***P<0.001 (Student’s *t*-test); NS=not significant. Data are representative of two (**g**,**h**,**i**,**j**,**k**), three (**c**,**e**) or four (**a**,**b**,**d**,**f**) experiments (mean±s.e.m., **f**,**g**,**h**,**i**,**j**,**k**). Immunoblot images have been cropped for presentation purpose. Full-size images are presented in Supplementary Fig. 3.

Recently, expression of CEACAM1 in granulocytes was shown to lead to Syk phosphorylation^37^. In addition, expression of CEACAM1 participates in Btk phosphorylation^38^. In B cells, Syk and Btk are essential signalling molecules, which are phosphorylated after BCR or BAFFR crosslinkage and play an essential role in induction of survival signals^15^,^39^. We thus focused on the influence of CEACAM1 on Syk activity during CEACAM1 and BCR crosslinkage. Indeed, in B-cell lymphoma immunoprecipitation of CEACAM1 pulled down Syk (Supplementary Fig. 2), which confirms the recently published interaction of Syk and CEACAM1 (ref. 37). To get insights into the functional relevance of CEACAM1 in B cells, we isolated splenocytes from WT and *Ceacam1*^*−/−*^ mice and challenged them with anti-IgM, anti-CEACAM1 activating antibody (monoclonal antibody, CC1) or lipopolysaccharide (LPS). Ig-α, which associated with BCR, showed slight tyrosine-phosphorylation after IgM crosslinkage (Fig. 2a, left panel). Neither anti-CEACAM1 monoclonal antibody challenge nor LPS treatment induced phosphorylation of Ig-α (Fig. 2a, right panel). We subsequently analysed Syk tyrosine phosphorylation in WT and *Ceacam1*^*−/−*^ mice. As expected BCR crosslinkage induced strong Syk phosphorylation. Absence of CEACAM1 strongly reduced Syk phosphorylation after BCR crosslinkage (Fig. 2b, left panel). Challenge with anti-CEACAM1 monoclonal antibody also triggered phosphorylation of Syk, which was stronger than LPS-induced Syk phosphorylation (Fig. 2b, right panel). Therefore, we concluded that CEACAM1 enhances Syk phosphorylation not only directly, but also after BCR crosslinkage. Next we analysed the downstream target of Syk, Btk, another member of the Tec family of non-transmembrane PTKs. Flow cytometric analysis showed that Btk is transiently phosphorylated in B cells derived from WT but not in *Ceacam1*^*−/−*^ mice after challenge with anti-IgM (Fig. 2c, upper panel) antibody. Similarly, anti-CEACAM1 monoclonal antibody challenge induced Btk phosphorylation in WT B cells (Fig. 2c, lower panel). We therefore conclude that CEACAM1 directly influences the phosphorylation of Syk and Btk, after BCR and CEACAM1 crosslinkage. To gain insight into downstream targets of CEACAM1 activation, we analysed specific Syk substrates. We observed that the CEACAM1 signal phosphorylate Erk1 (Erk-42) but not Erk2 (Erk-44) and, to a lesser extent, p-38. LPS could phosphorylate both Erk1/2 and p-38 (Fig. 2d,e). Triggering with the anti-CEACAM1 monoclonal antibody induced degradation of IκBα and slight phosphorylation of p65, a finding suggesting that CEACAM1 signals activate the canonical NF-κB pathway (Fig. 2f).

Tonic BCR signalling followed by Syk phosphorylation induces several survival genes, which are essential for B cell survival^12^. To see whether lack of CEACAM1 influences expression of those genes, we cultured purified splenic B cells from WT and *Ceacam1*^*−/−*^ mice for 24 h without further stimulation and measured mRNA expression of various NF-κB-regulated genes. Indeed, lack of CEACAM1 led to reduced expression of *Bcl6*, *Pax5*, *Bcl2a1* and *Xiap* (Fig. 2g). In line with the known positive feedback loop of NF-κB, we found reduced expression of *NF-κB* p65, *Rel-B*, *Nfatc1* and *Nfatc2* in *Ceacam1*^*−/−*^ B cells (Fig. 2h,i). *c-Jun*, *c-Fos* and *Ap1* were not influenced by CEACAM1 expression (Fig. 2j). *Blimp1*, which is another key regulator of plasma cell differentiation, was not affected by CEACAM1 expression (Fig. 2k). Taken together, we found that CEACAM1 contributed to the Syk phosphorylation during BCR and/or CEACAM1 crosslinkage, leading to the enhanced expression of NF-κB, NFAT and survival genes.

### CEACAM1 promotes survival of B cells *in vitro*

**Figure 3:**
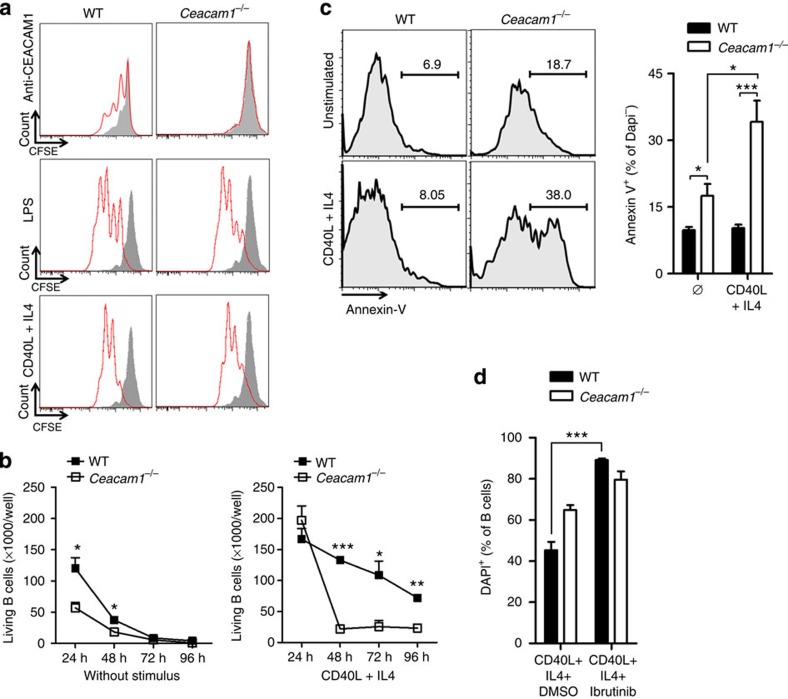
CEACAM1 promotes survival of B cells *in vitro*. (**a**) Representative flow cytometry histogram of proliferating B cells from wild-type (WT) or *Ceacam1*^*−/−*^ mice that were left untreated (grey area) or were further challenged (red line) with anti-CEACAM1 antibody, with LPS, or with recombinant mouse CD40 ligand in combination with mouse IL-4 for 48 h, as determined by FACS analysis. Histograms show DAPI^*−*^ cells (*n*=6). (**b**) Absolute numbers of living B cells (DAPI^*−*^) for indicated time points (*n*=3) sorted from spleen and after challenge with or without recombinant mouse CD40 ligand in combination with mouse IL-4 (*n*=3) determined by FACS analysis. (**c**) Representative histogram and statistical analysis of Annexin-V^+^ B cells, which were stimulated with recombinant mouse CD40 ligand in combination with mouse IL-4 or were left unstimulated for 48 h (gated on DAPI^***−***^ B cells, *n*=6). (**d**) Percentage of DAPI^+^ B cells sorted from spleen of WT and *Ceacam1*^*−/−*^ mice after challenge with recombinant mouse CD40 ligand in combination with mouse IL-4 cultured in the presence or absence of Btk inhibitor Ibrutinib for 48 h determined by FACS analysis (*n*=6). *P<0.05; **P<0.01 and ***P<0.001 (Student’s *t*-test). Data are representative of one of two (**b**) or two (**a**,**c**,**d**) experiments (mean±s.e.m. (**b**–**d**)).

We found that expression of CEACAM1 enhanced BCR-dependent Syk phosphorylation. Therefore, absence of CEACAM1 reduced expression of survival genes, which are induced via the tonic BCR signal. To see how lack of CEACAM1 influenced proliferation and survival of B cells, we labelled WT and *Ceacam1*^*−/−*^ B cells with carboxyfluorescein succinimidyl ester (CFSE) and challenged them with anti-CEACAM1 monoclonal antibody or the BCR-independent B-cell activator such as LPS, or CD40 ligand (CD40L) in combination with mouse interleukin (IL)-4. Challenge with anti-CEACAM1 (monoclonal antibody, CC1) induced some proliferation in WT but not in *Ceacam1*^*−/−*^ B cells (Fig. 3a). Challenge with LPS (TLR4-ligand) or CD40/IL-4 induced proliferation that was comparable between WT and *Ceacam1*^*−/−*^ B cells (Fig. 3a). Therefore, we conclude that the induction of proliferation via TLR4 or CD40 is not affected by CEACAM1.

Next, we analysed the impact of CEACAM1 on B-cell survival. First, we quantified the total number of living B cells with diamidino-2-phenylindole (DAPI) staining and fluorescence-activated cell sorting (FACS) beads. Without any further challenge, WT B cells consistently died under our *in vitro* culture conditions (Fig. 3b), a finding that agrees with that of a previous study^40^. Lack of CEACAM1 enhanced death of B cells (Fig. 3b). When challenged with CD40/IL-4, the numbers of WT B cells were higher than those of non-activated B cells after 24 h (Fig. 3b). The numbers of *Ceacam1*^*−/−*^ B cells 24 h after stimulation with CD40/IL-4 increased similarly to those of WT B cells, however, declined rapidly 24 h after challenge with CD40/IL-4 (Fig. 3b). This rapid decrease was probably due to apoptosis and cell death, because Annexin-V staining was significantly enhanced in none activated and activated DAPI^*−*^, B cells (Fig. 3c). We postulate that defective survival of *Ceacam1*^*−/−*^ B cells was due to limited Syk and Btk phosphorylation. To test this hypothesis, we treated WT and *Ceacam1*^*−/−*^ B cells with the Btk inhibitor Ibrutinib^41^. We observed that inhibition of Btk in WT B cells led to the rapid death of B cells. This effect was less pronounced in *Ceacam1*^*−/−*^ B cells (Fig. 3d). In conclusion, we found that lack of CEACAM1 signal limited survival of resting and activated B cells.

### CEACAM1 promotes B-cell differentiation and survival *in vivo*

**Figure 4:**
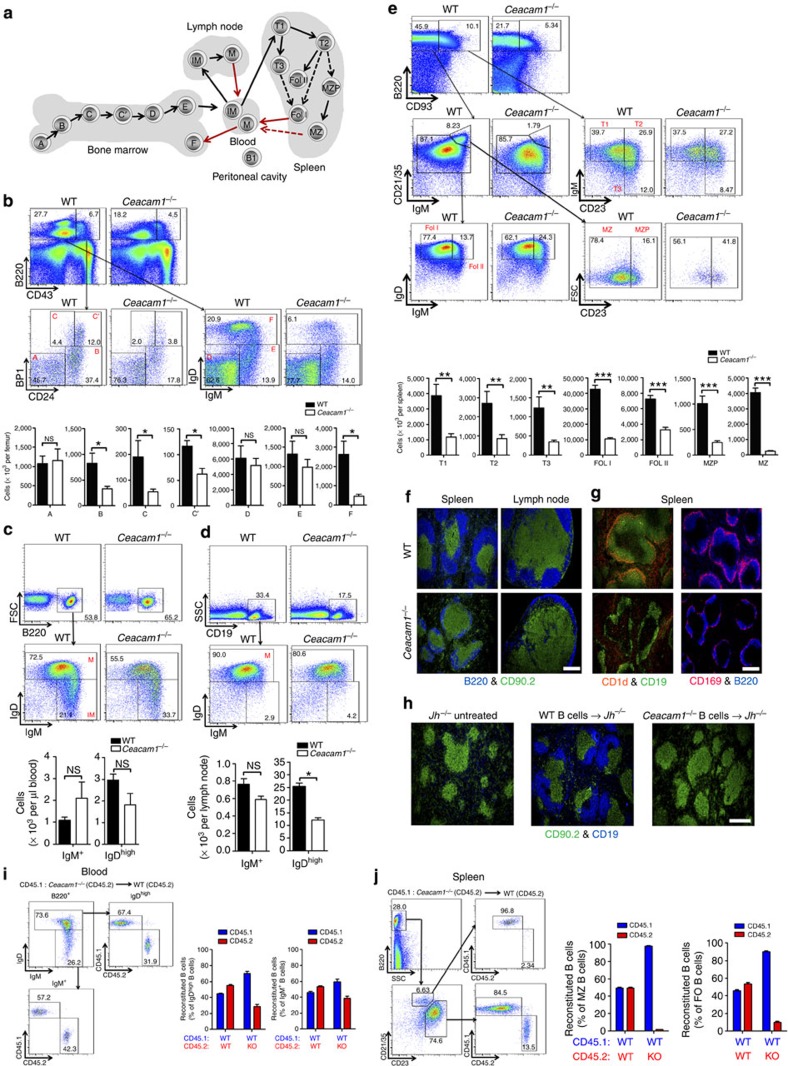
CEACAM1 promotes B-cell survival *in vivo*. (**a**) Scheme of developmental, maturation and migration stages of B cells in bone marrow, blood, lymph node and spleen. Arrows indicate most likely developmental pathway. Dotted arrow indicates still debated developmental pathway. Red arrow indicates differentiation after antigen challenge. (**b**–**e**) Representative dot blots, gating strategy and total numbers of B-cell subpopulations from wild-type (WT) and *Ceacam1*^*−/−*^ mice as measured by flow cytometry in bone marrow (**b**, *n*=6), in blood (**c**, *n*=4), in lymph node (**d**, *n*=4) and in spleen (**e**, *n*=10). (**f**,**g**) Representative immunofluorescence of spleen and lymph node (**f**, *n*=6) sections derived from naïve WT and *Ceacam1*^*−/−*^ mice after staining for B cells (B220, blue) or T cells (CD90.2, green) and spleen sections stained for marginal zone B cells (CD1d, red), follicular B cells (CD19, green), marginal zone macrophages (CD169, red) or B cells (B220, blue; **g**, *n*=6). (**h**) Representative immunofluorescence results of spleen sections from *Jh*^*−/−*^ mice that were left untreated or were subjected to adoptive transference with 1 × 10^7^ B cells derived from WT or *Ceacam1*^*−/−*^ mice 30 days before analysis, stained for T cells (CD90.2, green) and B cells (CD19, blue; *n*=3). (**i**,**j**) Representative dot blots, gating strategy and statistical analysis of B-cell subpopulations from murine bone marrow chimeras reconstituted with 1:1 composition of bone marrow from WT(CD45.1): WT(CD45.2) mice in one group and bone marrow from WT(CD45.1): *Ceacam1*^*−/−*^(CD45.2) mice in another group after 45 days of reconstitution (*n*=5 per group) as measured by flow cytometry in blood (**i**) and spleen (**j**; only second group has been shown for FACS gating strategy). Scale bars, 300 μm. *P<0.05; **P<0.01 and ***P<0.001 (Student’s *t*-test); NS=not significant. Data are representative of one (**h**) or two (**b**–**d**,**i**,**j**) or three (**e**) experiments (mean±s.e.m., **b**–**e**). One representative slide of three (**h**) or six (**f**,**g**) slides is shown.

We found that CEACAM1 is expressed on all B-cell subpopulations analysed and that it influences the survival of B cells, especially after activation. During maturation, several activating stimuli allow B cells to progress through various stages (Fig. 4a). To determine the influence of CEACAM1 on survival during B-cell proliferation, we analysed B-cell subsets in bone marrow, peripheral blood, lymph node and spleen of WT and *Ceacam1*^*−/−*^ mice. We found that B-cell precursor subsets in the bone marrow are reduced only in stages B, C and C′ (Fig. 4b). However, after stage C′ they expand more rapidly; therefore, the numbers of newly formed B cells in stages D and E are similar in WT and *Ceacam1*^*−/−*^ mice (Fig. 4b). This finding is concordant with the similar numbers of newly formed (immature, B220^+^IgM^+^) B cells in the peripheral blood (Fig. 4c). These findings are also in line with previously published experiments showing that there is no difference in B-cell numbers in the peripheral blood of *Ceacam1*^*−/−*^ mice^27^. Therefore, we conclude that the generation of newly formed B cells is hardly affected by CEACAM1. B-cell analysis in the peritoneal cavity showed that the frequency of B2 (CD19^+^B220^+^CD43^*−*^) B cells was slightly reduced. B1a (CD19^+^B220^+^CD43^+^CD5^+^) B-cell frequency was significantly reduced, whereas the B1b (CD19^+^B220^+^CD43^+^CD5^*−*^) B-cell population was higher in *Ceacam1*^*−/−*^ mice (Supplementary Fig. 4).

Next, we examined whether CEACAM1 signals are essential for B cells in secondary lymphoid organs, where newly formed B cells undergo further proliferation and differentiation. In lymph nodes, the numbers of newly formed (CD19^+^IgM^+^IgD^low^) B cells in WT and *Ceacam1*^*−/−*^ mice were similar (Fig. 4d). In contrast, the numbers of matured (M, CD19^+^IgD^high^) B cells were significantly reduced in the lymph nodes of *Ceacam1*^*−/−*^ mice (Fig. 4d). In the spleen, the T1, T2 and T3 populations of transitional B cells were reduced in the absence of CEACAM1 (Fig. 4e). The numbers of follicular B cells I (Fol I) and II (Fol II), MZ precursor cells and MZ B cells were dramatically reduced in the spleen of *Ceacam1*^*−/−*^ mice (Fig. 4e). In line with this finding, the numbers of mature B cells (stage F, B220^+^CD43^*−*^IgD^high^) in the bone marrow were reduced in *Ceacam1*^*−/−*^ mice (Fig. 4b). Similarly, histologic analysis showed lower numbers of B cells in the spleen and lymph node (Fig. 4f) and the absence of MZ B cells as well as low numbers of metallophilic (CD169^+^) macrophages in *Ceacam1*^*−/−*^ mice (Fig. 4g). To strengthen our hypothesis that reduced Syk phosphorylation after B-cell-activating factor receptor (BAFFR) or BCR activation, but not TLR or Fcgr2b signalling, is responsible for reduced B-cell numbers in *Ceacam1*^*−/−*^ mice, we performed histology of *Jh*^*−/−*^, *Baffr*^*−/−*^, *Fcgr2b*^*−/−*^ and *Myd88/Trif*^*−/−*^ mice. Histological analysis showed that a lack of BCR and BAFFR signalling, but neither TLR nor Fcgr2b signalling, reproduces the B-cell deficiency seen in *Ceacam1*^*−/−*^ mice (Supplementary Fig. 5).

Next, we determined whether defective intrinsic survival signals are responsible for reduced B-cell numbers in *Ceacam1*^*−/−*^ B cells, we transferred the same number of B cells from WT or *Ceacam1*^*−/−*^ mice into B-cell-deficient (*Jh*^*−/−*^) mice. Adoptively transferred WT B cells survived in the *Jh*^*−/−*^ mice and repopulated the spleen within 30 days (Fig. 4h). In contrast, *Ceacam1*^*−/−*^ B cells were not detectable after 30 days (Fig. 4h), a finding suggesting that the absence of intrinsic CEACAM1 limits the survival of B cells after proliferation in *Jh*^*−/−*^ mice. To gauge the direct influence of CEACAM1 expressed on B cells, we made use of mixed bone marrow chimeras, where the irradiation of WT (CD45.2) mice was followed by reconstitution with bone marrow from WT (CD45.1) and WT (CD45.2, mixed 1:1) or WT (CD45.1) and *Ceacam1*^*−/−*^ (CD45.2, mixed 1:1) mice. Analysis of peripheral blood showed some, but not statistically significant differenc in blood B-cell levels between WT and *Ceacam1*^*−/−*^ cells (Fig. 4i). In contrast, splenic MZ B cells were only derived from WT (CD45.1) mice (Fig. 4j). FO B cells were also mainly from WT (CD45.1) origin (Fig. 4j). This suggests that CEACAM1 B-cell intrinsic signals are essential for B-cell survival after B cells home to the spleen. Next, we analysed how the lack of CEACAM1 contributes to antibody production in unchallenged mice. Analysis of various immunoglobulin (Ig) subtypes in the serum of naïve WT and *Ceacam1*^*−/−*^ mice showed a reduction in IgM, IgG1, IgG2a, IgG2b, IgG3, IgA and IgE in *Ceacam1*^*−/−*^ mice (Supplementary Fig. 6). In conclusion, CEACAM1 expression influences survival in mature B cells.

### CEACAM1 ensures mouse survival during VSV challenge

**Figure 5:**
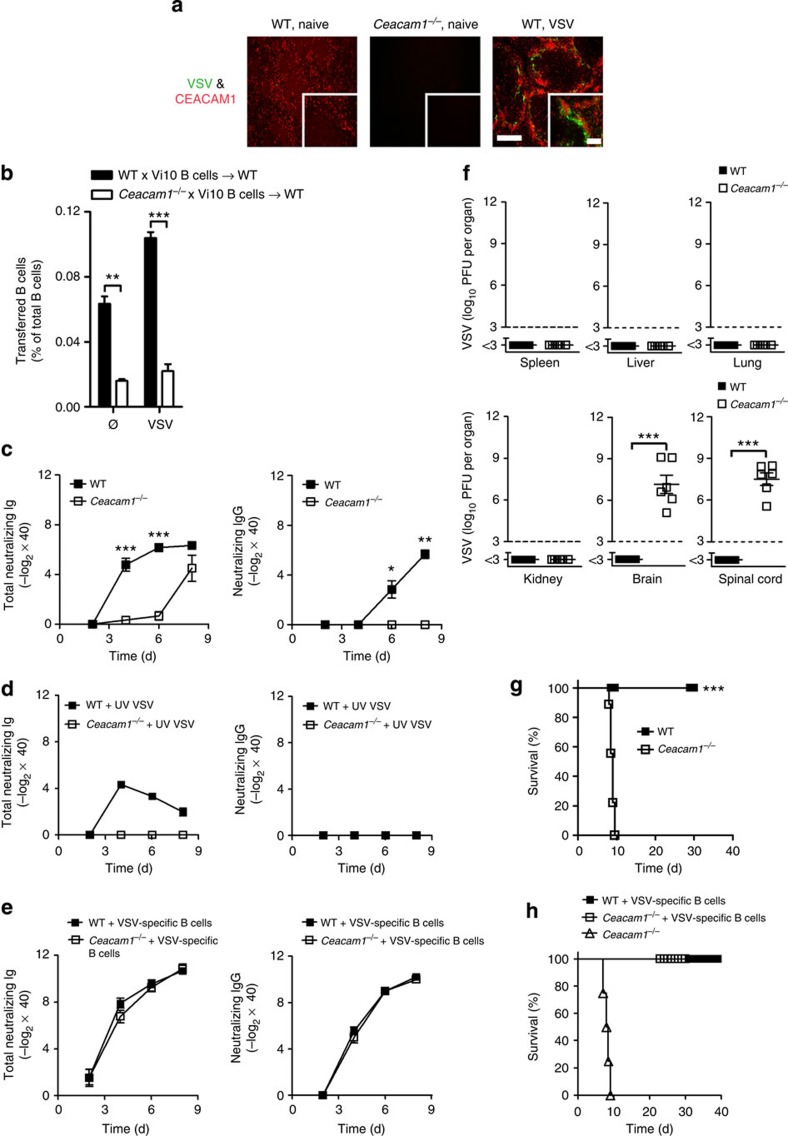
CEACAM1 ensures mouse survival during VSV challenge. (**a**) Immunofluorescence of spleen sections from naïve wild-type (WT) and *Ceacam1*^*−/−*^ mice and WT mice 7 h after infection with 2 × 10^8^ PFU of VSV (*n*=6), stained for VSV glycoprotein (green) and CEACAM1 (red). Scale bars, 300 μm (main image) or 100 μm (insets). (**b**) Bar diagram showing percentage of WT × Vi10 and *Ceacam1*^*−/−*^ × Vi10 B cells that were adoptively transferred into WT mice (1 × 10^7^ per mouse) on day −1 and which were infected with 2 × 10^6^ PFU of VSV on day 0. The proliferation was analysed in spleen 3 days after infection by FACS (*n*=3 per group). (**c**,**d**) Total VSV-neutralizing antibodies and neutralizing IgG antibodies generated in WT and *Ceacam1*^*−/−*^ mice after intravenous infection with 2 × 10^6^ PFU of VSV (**c**, *n*=6–9 per group) and/or after intravenous infection with 2 × 10^8^ PFU of ultraviolet (UV) light-inactivated VSV (**d**, *n*=8–9 per group). (**e**) VSV-neutralizing antibody response and neutralizing IgG antibodies measured in sera of WT and *Ceacam1*^*−/−*^ mice that also received 1 × 10^7^ VSV-specific B cells (Vi10) on day −1 and were then intravenously infected with 2 × 10^6^ PFU VSV on day 0 (*n*=7–9 per group). (**f**) VSV titres in various organs of WT and *Ceacam1*^*−/−*^ mice after intravenous infection with 2 × 10^6^ PFU of VSV assessed 8 days after infection (*n*=6 per group). (**g**) Survival of WT and *Ceacam1*^*−/−*^ mice after intravenous infection with 2 × 10^6^ PFU of VSV (*n*=9–12 per group). (**h**) Survival of WT and *Ceacam1*^*−/−*^ mice that were left untreated or were adoptively given 1 × 10^7^ VSV-specific B cells (Vi10) on day −1 and then intravenously infected with 2 × 10^6^ PFU of VSV on day 0 (*n*=4–9). *P<0.05; **P<0.01 and ***P<0.001 (Student’s *t*-test for **b**,**c**,**f**; Mentel-Cox survival test for **g** and **h**). Data are representative of one (**b**) or two (**a**,**f**) or three (**c**–**e**,**h**) or four (**g**) experiments (mean±s.e.m., **b**,**c**,**f**,**g**).

Next, we analysed the role of CEACAM1 during antigen challenge. We infected mice with VSV, a cytopathic pathogen for which systemic control strongly depends on the rapid induction of neutralizing antibodies^42^. We found strong expression of CEACAM1 in the MZ (Fig. 5a), a site at which VSV-specific B cells become activated^43^,^44^. Because CEACAM1 is a natural CEACAM1 ligand, this finding suggests that CEACAM1 may release an important survival signal during antigenic challenge. To determine the role of CEACAM1 during B-cell activation, we transferred same number of B cells from Vi10 mice (WT × Vi10), which express a VSV-specific BCR or from Vi10 mice crossed to *Ceacam1*^*−/−*^ mice (*Ceacam1*^*−/−*^ × Vi10), into WT mice and infected them with VSV. After 3 days, the number of WT × Vi10 B cells but not *Ceacam1*^*−/−*^ × Vi10 B cells was expanded (Fig. 5b). In the absence of stimulation, *Ceacam1*^*−/−*^ × Vi10 B cells were reduced as compared to WT × Vi10 B cells (Fig. 5b), a finding indicating that CEACAM1 provides an important survival signal in antigen-activated and non-activated B cells. In line with these data, *Ceacam1*^*−/−*^ mice displayed delayed induction of total VSV-neutralizing antibodies (Fig. 5c, left panel). In agreement with this finding, *Ceacam1*^*−/−*^ mice failed to generate neutralizing IgG antibodies (Fig. 5c, right panel). ELISA confirmed that VSV-specific IgG levels were significantly low in *Ceacam1*^*−/−*^ mice (Supplementary Fig. 7, right panel). Notably, anti-VSV IgM levels were significantly reduced in *Ceacam1*^*−/−*^ mice (Supplementary Fig. 7, left panel). During challenge with non-replicating ultraviolet-inactivated VSV, *Ceacam1*^*−/−*^ mice did not secrete neutralizing antibodies (Fig. 5d), a finding suggesting that *Ceacam1*^*−/−*^ mice exhibit strongly impaired B-cell functions. To demonstrate that CEACAM1 expression by B cells is responsible for poor VSV-neutralizing antibody responses, we adoptively transferred CEACAM1-competent VSV-specific B cells into WT and *Ceacam1*^*−/−*^ mice. This adoptive transfer of B cells rescued VSV-neutralizing antibody responses (Fig. 5e). Detailed analysis showed that both MZ and FO B cells could rescue the VSV-neutralizing antibody response in the *Ceacam1*^*−/−*^ mice (Supplementary Fig. 8). Absence of early neutralizing antibodies can result in the spread of VSV to the nervous system^45^. To determine whether a reduction in the levels of neutralizing antibodies in *Ceacam1*^*−/−*^ mice affected the immune response of virus control, we analysed organ virus titres 8 days after systemic VSV infection. Indeed, *Ceacam1*^*−/−*^ mice exhibited VSV replication in the brain and spinal cord, whereas WT mice controlled VSV replication in all organs (Fig. 5f). VSV replication in *Ceacam1*^*−/−*^ brains was associated with the death of mice (Fig. 5g). To determine whether a diminished B-cell response contributed to mortality, we performed survival experiments. *Ceacam1*^*−/−*^ mice with adoptively transferred VSV-specific WT B cells survived VSV infection (Fig. 5h). Therefore, B-cell intrinsic expression of CEACAM1 is essential for initiating a protective antiviral immune response after exposure to VSV.

### CEACAM1 facilitates LCMV-dependent B-cell activation

**Figure 6:**
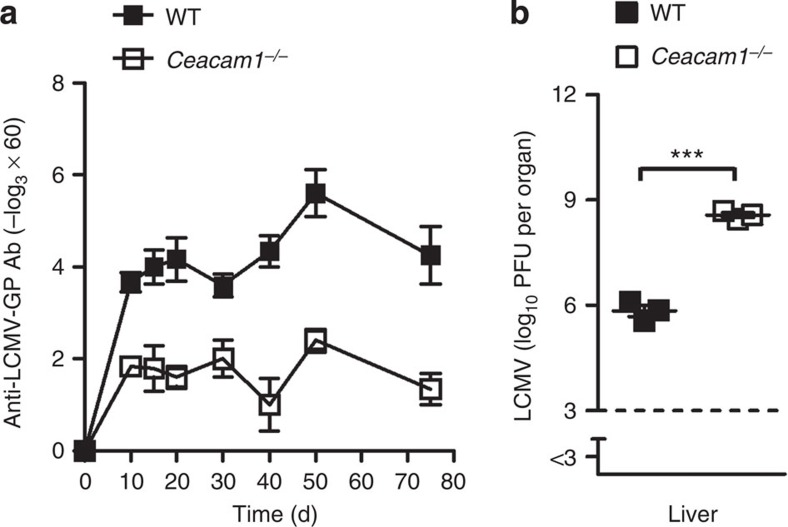
CEACAM1 facilitates LCMV-dependent B-cell activation. LCMV glycoprotein-specific IgG antibodies measured in sera of wild-type (WT) and *Ceacam1*^*−/−*^ mice after intravenous infection with 200 PFU of LCMV-WE (*n*=3–6 per group). (**b**) Liver virus titres of WT and *Ceacam1*^*−/−*^ mice 10 days after intravenous infection with 2 × 10^6^ PFU of LCMV-WE (*n*=3 per group). ***P<0.001 (Student’s *t*-test). Data are representative of one (**b**) or two (**a**) experiments (mean±s.e.m., **a**,**b**).

Next, we infected WT and *Ceacam1*^*−/−*^ mice with non-cytopathic lymphocytic choriomeningitis virus (LCMV) to confirm whether antigen-specific B-cell responses are impaired in *Ceacam1*^*−/−*^ mice. Early control of an infection dose of 200 plaque-forming units (PFUs) of LCMV depends only on CD8^+^ T cells. However, at higher infection doses, B cells are important for LCMV control^46^. WT mice produce higher titres of LCMV-specific antibodies than do *Ceacam1*^*−/−*^ mice during LCMV infection (Fig. 6a). In line with this finding, LCMV replication in the liver of *Ceacam1*^*−/−*^ mice was prolonged (Fig. 6b).

### Human B-cell subpopulations express CEACAM1

**Figure 7:**
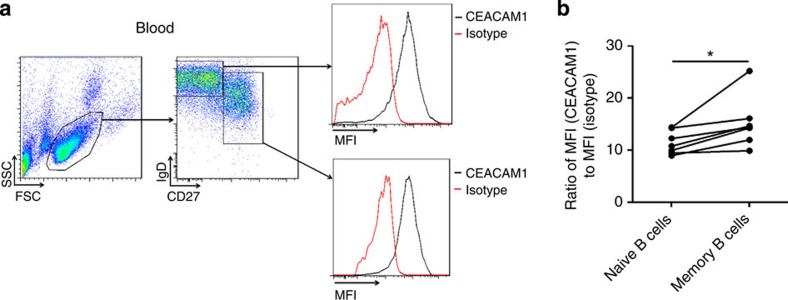
Human B-cell subpopulations express CEACAM1. (**a**) Representative FACS plot and histogram showing CEACAM1(monoclonal antibody, B3-17) expression in lymphocyte subpopulations in human peripheral blood on naive (IgD^+^CD27^*−*^) and memory (CD27^+^) B cells. Isotype control antibody staining of lymphocytes is shown as red line (*n*=6). (**b**) Histogram plot showing differences in mean fluorescence intensity (MFI) levels between human naïve and memory B cells (*n*=6). *P<0.05 (Student’s *t*-test). Data are representative of independent staining from six donor samples (mean±s.e.m., **b**).

To determine whether CEACAM1 also plays a potential role in human B cells, we measured CEACAM1 expression by naïve B cells. Previous publications reported that most B cells isolated from peripheral blood express CEACAM1 (ref. 32). Also, we found that naive human B cells (IgD^high^CD27^*−*^) and memory B cells (CD27^+^) express substantial levels of CEACAM1 (Fig. 7a). The expression of memory B cells is slightly higher than that of naive B cells (Fig. 7b), a finding suggesting that the CEACAM1 signal in humans may be crucial for the survival of memory B cells rather than naïve B cells.

## Discussion

The results of this study demonstrate that CEACAM1 expression is essential for the survival of murine B cells. The absence of CEACAM1 expression on murine B cells is associated with reduced numbers of B cells and a defective immune response after viral antigen challenge.

Greicius *et al*.^31^ demonstrated that binding of CEACAM1 with an anti-CEACAM1 monoclonal antibody induces strong B-cell proliferation. Other studies showed that BCR activation in the presence of another anti-CEACAM1 monoclonal antibody (clone T84.1) limits B-cell proliferation^47^,^48^. In the current study, we demonstrate that CEACAM1 crosslinkage had some effect on proliferation. However, *in vitro* and *in vivo* we found a strong influence of CEACAM1 on the survival of activated B cells. Therefore, we consider that a prolonged signal via CEACAM1 ligation is essential for promoting survival signals and maintaining B-cell numbers, which could explain reduced B-cell numbers in *Ceacam1*^*−/−*^ mice.

BCR crosslinking by auto-antigens has an essential role in the differentiation of transitional B cells into MZ B cells and follicular B cells^6^. In addition, this tonic BCR signal, together with constant signalling via the BAFF receptor, is the most important survival signal for naïve B cells. How this diverse process can be regulated by BCR signals remains unexplained. We suggest that CEACAM1 is another strong regulator of B-cell survival. This is supported by previous findings that CEACAM1 regulates apoptosis in granulocytes^49^. Because CEACAM1 is expressed most strongly by B cells, and because CEACAM1 is its own ligand, we suggest that CEACAM1 induces positive survival signals primarily on B cells within the B-cell follicles and that CEACAM1 acts as a positive feedback loop once B cells reach the B-cell follicle. Therefore, in addition to chemokine gradients^50^, CEACAM1 expression appears to contribute to the generation of B-cell follicles.

We found that Syk phosphorylation after BCR crosslinkage was dependent on expression of CEACAM1. In line with these results, monoclonal antibody activation of CEACAM1 led to Syk phosphorylation. These data suggest that CEACAM1 directly interacts with BCR signalling and therefore would influence the tonic BCR signal as well as antigen-dependent BCR activation. Tonic BCR signalling, but also BAFFR signalling, induces several survival genes via Syk. Therefore, lack of BCR and BAFFR signalling limits B-cell development and survival. Indeed, in line with data from *Jh*^*−/−*^ or *Baffr*^*−/−*^ mice, *Ceacam1*^*−/−*^ mice showed strongly reduced B-cell numbers in naïve mice. Therefore, we concluded that CEACAM1, in addition to BAFFR, is another important membrane molecule influencing BCR signalling via Syk.

We found that CEACAM1 activation leads to Btk phosphorylation. This finding is related to the enhanced survival of B cells. Loss-of-function mutations in BTK lead to X-linked agammaglobulinemia because of a complete absence of mature B cells^51^. Mice deficient in CD19 or BCR exhibit a strongly reduced number of mature B cells^52^. This reduction in the numbers of MZ B cells in CEACAM1-deficient mice is also found in mice and humans lacking the Wiskott-Aldrich syndrome protein (WASp)^53^. WASp is also involved in BTK phosphorylation^54^. As we have shown for CEACAM1, the necessity for WASp in the generation of the MZ B-cell compartment is B-cell intrinsic^55^. Whether CEACAM1 affects WASP function must be investigated in future studies.

We found that CEACAM1 is expressed on human B cells. This could suggest that also in human antiviral immune response, CEACAM1 signalling may play a role in inducing sufficient antibody responses against different viruses. Therefore, we would suggest that lack of CEACAM1 could be another factor explaining B-cell deficiency in humans. If indeed CEACAM1 deficiency occurs in humans, remains to be fully elucidated.

Recently, we found that CD169^+^ macrophages enforce viral replication and therefore are essential for initiating an antiviral immune response^44^,^56^. B cells play an important role in recruiting CD169^+^ macrophages^3^. Therefore, B-cell deficiency leads to defects in enforced virus replication (Supplementary Fig. 9) and a reduction in the innate immune response. Indeed, we found that in *Ceacam1*^*−/−*^ mice the ability of CD169^+^ macrophages to enforce virus replication was impaired (Supplementary Fig. 10). Which could be an additional mechanism how CEACAM1 deficiency in B cells contributes to defective anti-VSV immune response.

In conclusion, we found that CEACAM1 expressed on murine B cells is an important regulator of B-cell homeostasis. During exposure with cytopathic virus, CEACAM1 was essential for inducing an efficient antiviral antibody response and thereby reducing mouse mortality.

## Methods

### Mice

All mice used in this study, including *Ceacam1*^*−/−*^, *Jh*^*−/−*^, *Tcrab*^*−/−*^, *Aid*^*−/−*^, *sIgM*^*−/−*^, *Myd88/Trif*^*−/−*^, *Baffr*^*−/−*^ and *Fcgr2b*^*−/−*^ mice, were maintained on the C57BL/6 genetic background (back-crossed at least 8 times and as many as 16 times) and were bred as homozygotes. Vi10/CD45.1 mice expressing VSV-specific BCR as a transgene were used for cell transfer studies, and mice expressing the CD45.1 transgene were used for reconstitution of bone marrow. Six- to eight-week-old, age- and sex-matched mice were used for all the studies. All animals were housed in single ventilated cages. During survival experiments, the health status of the mice was checked twice daily. Animal experiments were authorized by the Nordrhein Westfalen Landesamt für Natur, Umwelt und Verbraucherschutz (Recklinghausen, Germany), and in accordance with the German law for animal protection or according to institutional guidelines at the Ontario Cancer Institute of the University Health Network and at McGill University. Animals exhibiting severe symptoms of sickness or paralysis or showing substantial weight loss during VSV infection were put to death and were considered dead for statistical analysis.

### Bone marrow chimeras

For generation of bone marrow chimeras, C57BL/6 mice were irradiated twice for 7 min each with a total of 1,050 rad. After 24 h, mice were reconstituted intravenously with 5 × 10^6^ bone marrow cells from each donor for mixed bone marrow chimeras. Mice were analysed 40–45 days after reconstitution.

### Virus and plaque assays

VSV, Indiana strain (VSV-IND, Mudd-Summers isolate), was originally obtained from Professor D. Kolakofsky (University of Geneva, Switzerland). Virus was propagated on BHK-21 cells at a multiplicity of infection of 0.01. VSV concentration was determined as described below, and was then plaque-purified on Vero cells^57^. Mice were infected intravenously with VSV at the indicated doses. Virus titres were measured with a plaque-forming assay. For this assay, organs were smashed in Dulbecco’s modified Eagle medium (DMEM) containing 2% fetal calf serum (FCS), titrated 1:3 over 12 steps, and plaqued on Vero cells. After a 2-h incubation at 37 °C, overlay was added, and the virus preparation was again incubated at 37 °C. Plaques were counted 24 h later by the use of crystal violet staining. The LCMV strain WE was originally obtained from F. Lehmann-Grube (Heinrich Pette Institute, Hamburg, Germany) and was propagated on L929 cells, MC57 cells or both. Mice were infected intravenously at the indicated dose. LCMV viral titres were detected by a plaque-forming assay on MC57 fibroblasts as previously described^58^. In short, smashed organs were plaqued with MC57 cells as described above and incubated at 37 °C. After a 3-h incubation at 37 °C, overlay was added, and the virus preparation was again incubated at 37 °C. Plaques were counted 72 h later by LCMV NP staining. Cells were fixed (with 4% formaldehyde solution), permeabilized (with 1% Triton-X solution), blocked (with 10% FCS in phosphate-buffered saline) and stained with anti-LCMV NP antibody (made in house). ECL-conjugated anti-rabbit-IgG antibody was used as a secondary antibody. Plaques were detected by colour reaction (0.2 M Na_2_HPO_4_+0.1 M citric acid+30% H_2_O_2_+*o*-phenylenediamine dihydrochloride), all chemicals from Sigma-Aldrich.

### Neutralizing antibody assay

Serum was prediluted (1:40). The complement system was inactivated at 56 °C for 30 min. For analysis of IgG kinetics, diluted samples were treated with 2-mercaptoethanol (0.1 M) for removal of IgM. Serum was titrated 1:2 over 12 steps and was incubated with 1 × 10^3^ PFU of VSV. After a 90-min incubation at 37 °C, the virus–serum mixture was plaqued onto Vero cells. Overlay was added after 1 h, and the mixture was incubated again for 24 h at 37 °C. Plaques were counted by crystal violet staining. Antibody titres are presented as two- or threefold dilution steps (−log_2_ and −log_3_) times the predilution (that is, × 40).

### B-cell culture

Spleens retrieved from WT and *Ceacam1*^*−/−*^ mice were homogenized in magnetic-activated cell sorting (MACS) buffer (1% FCS and 0.8% 0.5 M EDTA). B220^+^ B cells were isolated by positive selection with CD45R-conjugated magnetic beads (MACS Miltenyi Biotech). Flow cytometry confirmed that the purity of B220^+^ cells was higher than 95%. For proliferation assays, cells were labelled with 5 μM carboxyfluorescein succinimidyl ester, and 2 × 10^5^ cells per well were cultured in 96-well flat bottom plates in RPMI 1640 medium supplemented with 10% LPS-free FCS, 1% antibiotics and 0.1% 50 mM 2-mercaptoethanol. They were then challenged with anti-CEACAM1 (20 μg ml^−1^, clone CC1; a kind gift from Dr Kathryn V. Holmes, University of Colorado, Denver, CO) antibody or recombinant mouse CD40 ligand (1 μg ml^−1^) in combination with mouse IL-4 (10 ng ml^−1^; R&D Systems) or LPS (100 ng ml^−1^; Sigma-Aldrich) for 48 h. Similarly, for inhibition experiments, purified B cells (as described above) were cultured in the medium described above with recombinant mouse CD40 ligand (1 μg ml^−1^) in combination with mouse IL-4 (10 ng ml^−1^; R&D Systems) and were treated with 10 μM Ibrutinib (Selleck). Control wells were supplemented with equal amounts of DMSO used to dissolve Ibrutinib. Cell death was measured by DAPI (Life Technologies) staining. For survival experiments, B cells were cultured as mentioned above, and cells were stained with Annexin-V (BD Biosciences) followed by DAPI. Proliferation and survival were determined by FACS at indicated time points. For cell signalling experiments, splenocytes were dissociated in VLE-DMEM supplemented with 10% LPS-free FCS and 1% antibiotics and were challenged with anti-CEACAM1 (clone CC1, 20 μg ml^−1^; K. Holmes) and anti-IgM (10 μg ml^−1^) antibody (Jackson ImmunoResearch Laboratories, Inc.) for various time periods at 37 °C.

### Histology

Histologic analyses of snap-frozen tissue were performed with a monoclonal antibody to VSV glycoprotein (Vi10; made in-house). Anti-CD45R (B220; RA3-6B2), anti-CD90.2 (53-2.1), anti-CD19 (1D3), anti-CD1d (1B1) and anti-CEACAM1 (CC1) monoclonal antibodies were purchased from eBioscience. Biotin-anti-CD169/SIGLEC1 (MOMA-1) was purchased from Acris. Sections were washed and stained with streptavidin (eBioscience). In short, sections were fixed with acetone, and nonspecific antigens were blocked in PBS containing 2% FCS for 15 min, followed by various stainings of antibodies, diluted 1:100 in blocking solution for 45 min. Images of stained sections were acquired with a fluorescence microscope (KEYENCE BZ II analyzer).

### Flow cytometry

Peripheral blood cells were stained with anti-Ly6G (RB6-8C5), anti-CD115 (AFS98), anti-CD45R (B220; RA3-6B2), anti-CD90.2 (30-H12), anti-CEACAM1 (CC1; with corresponding isotype control anti-IgG1 (M1-14D12)), anti-IgD (11-26c), anti-CD93 (AA4.1), anti-CD19 (1D3) (all from eBioscience) and anti-IgM (II/41; BD Biosciences) antibodies. Recovered bone marrow cells were resuspended in FACS buffer (0.5 M EDTA, 0.1% sodium azide, 1% FCS in PBS) and incubated with anti-CD45R (B220; RA3-6B2), anti-IgD (11-26c), anti-IgM (II-41), anti-CEACAM1 (CC1), anti CD24 (M1/69; all from eBioscience), anti-CD43 (1B-11), and anti-BP1 (6C3; from BioLegend) antibodies. Inguinal lymph nodes were disaggregated in FACS buffer, and cells were stained for anti-CD45R (B220; RA3-6B2), anti-CD19 (1D3), anti-IgD (11-26c; all from eBioscience), and anti-IgM (II/41; BD Biosciences) antibodies. Spleens were dissociated in FACS buffer, and splenocytes were incubated with anti-CD45R (B220; RA3-6B2), anti-CD19 (1D3), anti-CD93 (AA4.1), anti-CD21/35 (8D9), anti-CD23 (B3B4), anti-IgD (11-26c), anti-CEACAM1 (CC1), anti-CD45.1 (A20), anti-CD45.2 (104; all from eBioscience) and anti-IgM (II/41; BD) antibodies. Human peripheral blood samples were stained with anti-IgD (IA6-2) and anti-CD27 (M-T271) antibodies (both from BD Biosciences) and anti-CEACAM1 (monoclonal antibody, B3-17) antibody (from Dr Singer, Essen). Peritoneal B cells were stained for anti-CD19 (1D3), anti-CD45R (B220; RA3-6B2), anti-IgM (II/41; all from eBioscience), and anti-CD5 (53-7.3; BD Biosciences) and anti-CD43 (1B-11; from BioLegend) antibodies. Dead cells were discriminated by staining with propidium iodide (PI, eBioscience) and/or DAPI and were excluded from all analyses except for blood. For cell signalling experiments, cells were fixed and permeabilized according to the manufacturer’s instructions (BD Phosflow, BD Biosciences). The cells were stained with anti-Btk (pY223)/Itk (pY180) (BD Phosflow) antibody. All antibodies were diluted 1:100 to their original concentration in FACS buffer. For quantification of total cell numbers, FACS beads were used (BD Biosciences). All stained cells were analysed on an LSRII or a FACSFortessa (BD Biosciences) flow cytometer, and data were analysed with Flowjo software.

### Immunobloting

Spleens were dissociated, and splenocytes were subjected to erythrocyte lysis buffer (MORPHISTO). Next, 10 × 10^6^ cells were challenged with or without anti-CEACAM1 (clone CC1, 20 μg ml^−1^) antibodies, anti-IgM (Jackson ImmunoResearch Laboratories, Inc., 10 μg ml^−1^) antibodies, and LPS (Sigma-Aldrich, 100 ng ml^−1^) treatment for indicated time points. Cells were lysed with boiling SDS buffer (1.1% SDS, 11% glycerol, 0.1 M Tris; pH 6.8) with 10% 2-mercaptoethanol. Total cell extracts were examined by 10% SDS–PAGE gels and transferred onto Whatman nitrocellulose membrane (GE Healthcare) by standard techniques. Membranes were blocked for 1 h in 5% BSA (PAA Laboratories) in TBS supplemented with 1% Tween-20 and incubated with the following antibodies: anti-phospho-Igα (Y182); anti-phospho-Syk/ZAP (Y352/Y319); anti-phospho-p44/42 (p-Erk1/2); anti-phopshpo-p38; anti-phospho-NF-κB p65; anti-Syk; anti-p44/42 (Erk1/2), anti-p38; anti-IκBα (all from Cell Signalling Technologies); anti-Igα (Thermo Scientific); and anti-NF-κB p65 (Santa Cruz). The secondary antibodies anti-actin (Cell Signalling Technologies) and anti-GAPDH (Meridian Life Science) were detected by horse radish peroxidase (HRP)-conjugated anti-mouse IgG (Bio-Rad) or anti-rabit IgG (GE Healthcare) antibodies, or both. Signals were detected with the BIO RAD ChemiDoc imaging system and analysed with the manufacturer’s software. Blots were quantified with KODAK MI software. Images have been cropped for presentation purpose. Full-size images are presented in Supplementary Fig. 3.

### Immunoprecipitation

Mouse B-lymphocyte H16-L10-4R5 cells were cultured in RPMI 1660 medium supplemented with 10% FCS and 1% antibiotics. 25 × 10^6^ cells were lysed in dulbecco's phosphate-buffered saline (DPBS) supplemented with 1% Triton-X and protease inhibitor cocktail (both from Sigma-Aldrich). Lysates were incubated with anti-CEACAM1 antibody (CC1, Novus Biologicals, 5 μg ml^−1^) overnight at 4°. CEACAM1 was immunoprecipitated with protein G dynabeads (Life technologies).

### RT–PCR

Total RNA was extracted from MACS-sorted pure B cells with TRIZOL reagent (Ambion) according to the manufacturer’s instructions. RNA was transcribed with a QuantiTect Reverse Transcription Kit (Qiagen). Quantitative real-time PCR amplification of single genes was performed with SYBR Green quantitative PCR master mix in a Light Cycler 480 (Roche). QuantiTect Primer assays for Bcl-6, Pax-5, Bcl2a1, Xiap, NF-κB, Rel-B, Nfatc1, Nfatc2, c-Jun, c-Fos, Ap1S1 and Blimp-1 (Qiagen) were used for quantification of mRNA expression of the respective genes. The following oligonucleotide primers that detect murine CEACAM1 isoforms were used for detection: mouse CEACAM1, a common sense primer that detects both long and short isoform FP-5′- GCCATGCAGCCTCTAACCCACC -3′; and two antisense primers that detect specific isoforms, mouse CEACAM1-L BP-5′- CTGGAGGTTGAGGGTTTGTGCTC -3′ and mouse CEACAM1-S BP-5′- TCAGAAGGAGCCAGACCCGCC -3′. The product was analysed on 3% agarose gels in Tris-borate-EDTA buffer and visualized by ethidium bromide staining. For analysis, expression levels of target genes were normalized to glyceraldehyde 3-phosphate dehydrogenase, 18S r-RNA (Qiagen), or both as an internal control gene (ΔCt). Gene expression values were then calculated with the ΔΔCt method; the mean of four untreated WT B cells (0 h after sorting) was used as a calibrator to which all other samples were compared. Relative quantities (RQs) were determined with the equation *RQ*=2^*−*ΔΔCt^.

### LCMV-glycoprotein GP1-specific IgG measurements

The detection of LCMV-glycoprotein GP-1-specific IgG by ELISA has been previously described^59^. In short, 96-well flat-bottom Nunc Immuno Plates (Thermo Scientific) were coated with anti-human IgG (Jackson ImmunoResearch Laboratories, Inc.) in coating buffer (0.1 M Na_2_CO_3_+0.1 M NaHCO_3_; pH 9.6) overnight at 4 °C. On the next day, plates were washed with washing buffer (PBS with 0.05% Tween-20), and unspecific binding was blocked with 2% FCS in PBS for 2 h. Plates were incubated with LCMV Gp-Fc supernatant (made in-house) for 3 h at room temperature. Plates were washed and titrated with pre-diluted (1: 20) serum over 12 wells with 1: 3 dilutions in successive wells. After a 90-min incubation, plates were incubated with HRP-conjugated anti-mouse-IgG antibody (Sigma). After a 1-h incubation, plates were developed as described below.

### ELISA measurements

For detection of VSV-specific anti-IgG and anti-IgM antibodies, 96-well flat-bottom Nunc Immuno Plates (Thermo Scientific) were coated with baculovirus VSV-GP^42^ in coating buffer 0.1 M NaCO_3_ (0.1 M Na_2_CO_3_+0.1 M NaHCO_3_; pH 9.6) overnight at 4 °C. On the next day, plates were washed with washing buffer (PBS with 0.05% Tween-20), and unspecific binding was blocked with 2% FCS in PBS for 1–2 h. Plates were washed once and titrated with prediluted (1:15) serum over 12 wells with 1:3 dilutions in successive wells. Plates were incubated at room temperature for 2 h. Plates were washed with washing buffer and incubated with HRP-conjugated anti-mouse-IgG (Sigma) or anti-mouse-IgM (Sigma) antibody for 30–60 min. Plates were washed and incubated with 1 × TMB Substrate solution (eBioscience) in the dark, after which 10% H_2_SO_4_ solution was added to stop the colour reaction. Optical density was measured at 450 nm (FLUOstar Omega, BMG LABTECH). Various immunoglobulin isotypes and subtypes were measured in naïve serum of WT and *Ceacam1*^*−/−*^ mice as described^55^.

### Statistical analysis

Data are expressed as mean±s.e.m. Student’s *t*-test was used to detect statistically significant differences between groups. Significant differences between several groups were detected by one-way analysis of variance (ANOVA) with Bonferroni or Dunnett *post hoc* tests. Survival was compared with log-rank (Mantel-Cox) tests. The level of statistical significance was set at *P*<0.05.

## Author contributions

V.K. and V.D. designed, planned and performed the experiments, analysed data and wrote the paper. S.K.M. helped in experiments and was involved in data analysis. N.H. and N.S. were involved in data discussion. A.P. helped in experiments. M.S. performed studies on human lymphocytes. V.P., H.C.X. and P.S. helped in experiments. F.B. and F.M. performed immunoglobulin isotype and subtype ELISAs. K.M. and E.L. helped in experiments. C.K., A.M.W., D.H., F.L., U.D., R.K., M.R., C.H. and I.S. were involved in the data discussion and in drafting the manuscript. N.B. provided the *Ceacam1*^*−/−*^ mice and was involved in the data discussion and in drafting the manuscript. J.R.G. performed the B-cell analysis, provided the reagents, discussed the data and wrote the paper. B.B.S. initiated the study, provided reagents, organized and discussed the data and wrote the paper. P.A.L. discussed the data and wrote the paper. K.S.L. initiated, organized and designed the study, wrote the paper and completed the manuscript.

## Additional information

**How to cite this article:** Khairnar, V. *et al*. CEACAM1 induces B-cell survival and is essential for protective antiviral antibody production. *Nat. Commun.* 6:6217 doi: 10.1038/ncomms7217 (2015).

## Supplementary Material

Supplementary Information Supplementary Figures 1-10
