# Supplementary material for: CEACAM1 induces B-cell survival and is essential for protective antiviral antibody production
Source: Nat Commun. 2015 Feb 18;6:6217. doi: 10.1038/ncomms7217 (PMC4346637; doi:10.1038/ncomms7217)
Supplement: Supplementary Information — Supplementary Figures 1-10 [file ncomms7217-s1.pdf]

Supplementary Figure 1

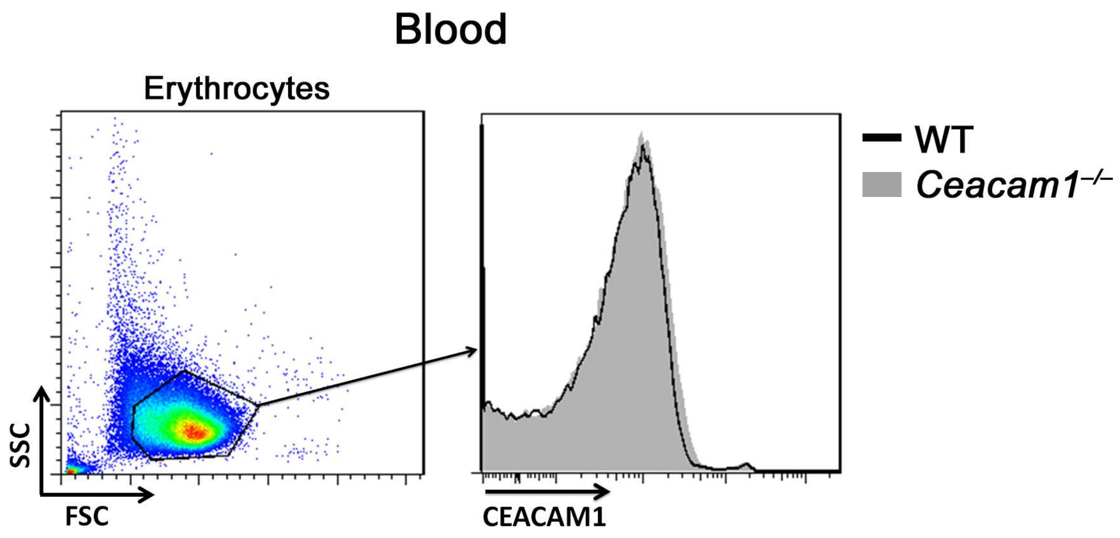

Supplementary Figure 1: Erythrocytes stain negative for CEACAM1
Representative dot plot and histogram showing CEACAM1 expression in peripheral blood erythrocytes from wild-type (WT, black line) and *Ceacam1*$^{-/-}$ mice (grey area) as measured by flow cytometry (n = 3−4 per group).

Suplementary Figure 2

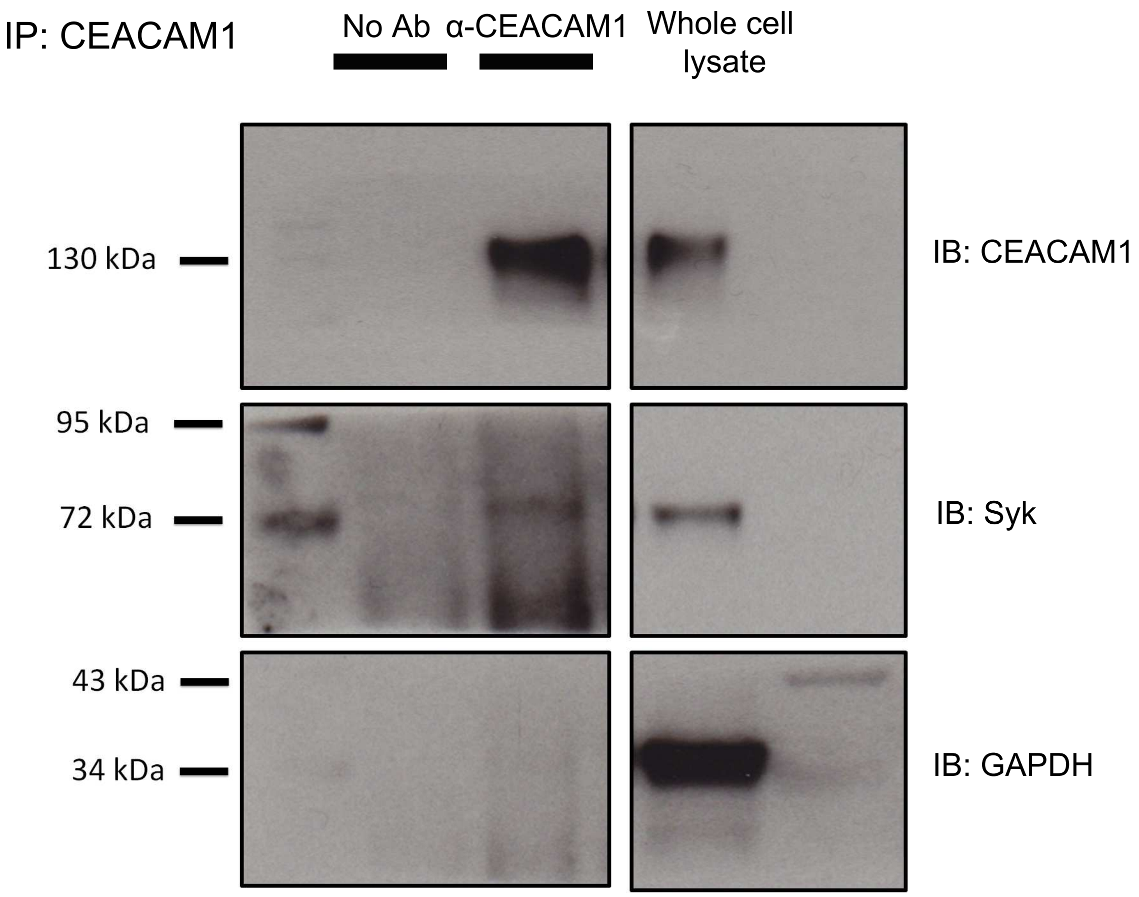

Supplementary Figure 2: CEACAM1 interacts with Syk
Immunoblot analysis of CEACAM1, Syk and GAPDH in mouse B-lymphocyte from whole cell lysate and after immunoprecipitation with or without anti-CEACAM1 antibody (mAb, CC1). Data are representative of three independent experiments.

Supplementary Figure 3

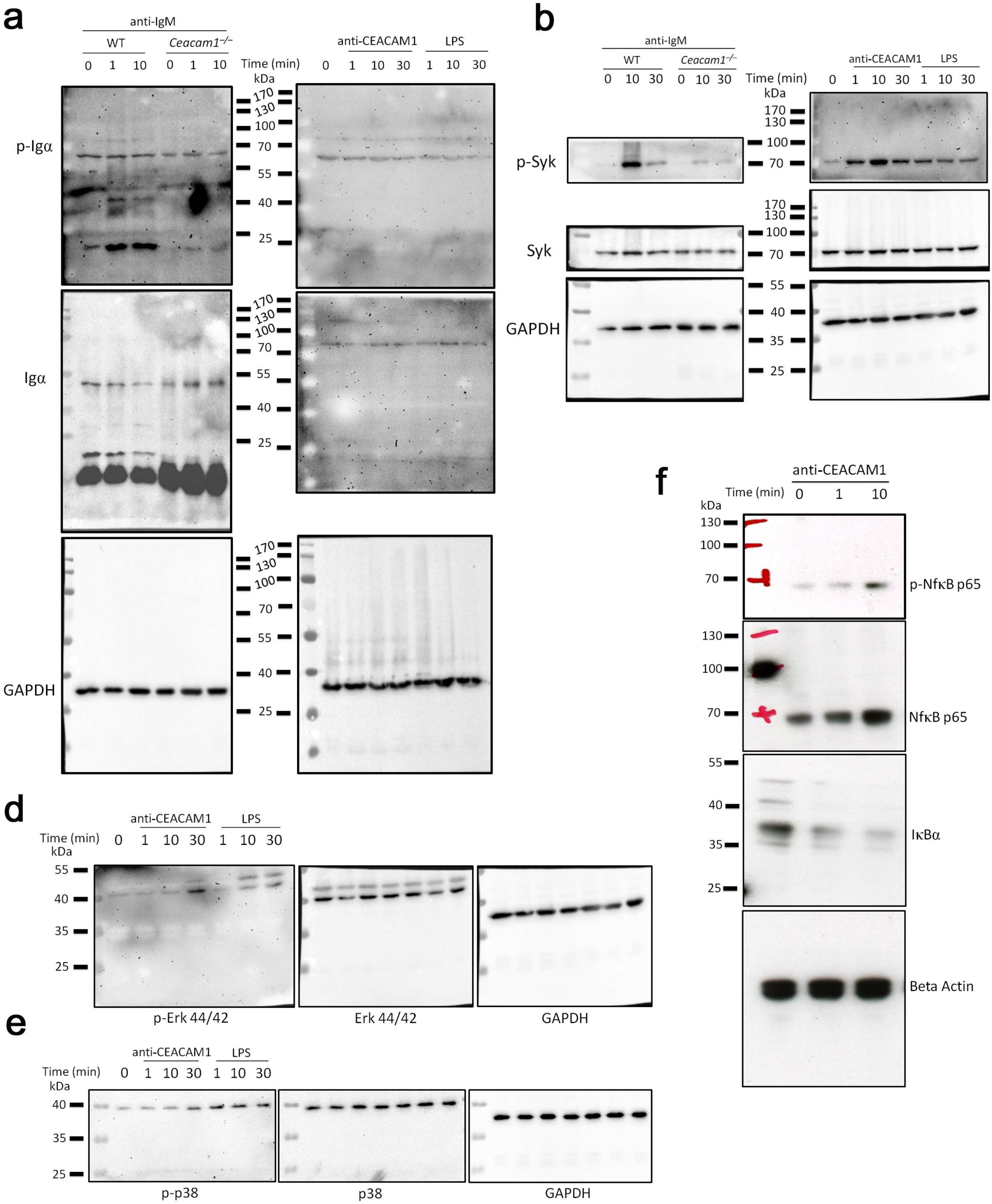

Supplementary Figure 3: Uncropped western blots shown in Figure 2. Proteins were loaded on 10% SDS-PAGE gels and transferred onto Whatman nitrocellulose membrane by standard techniques. Nitrocellulose membranes were subsequently developed from left to right.

Supplementary Figure 4

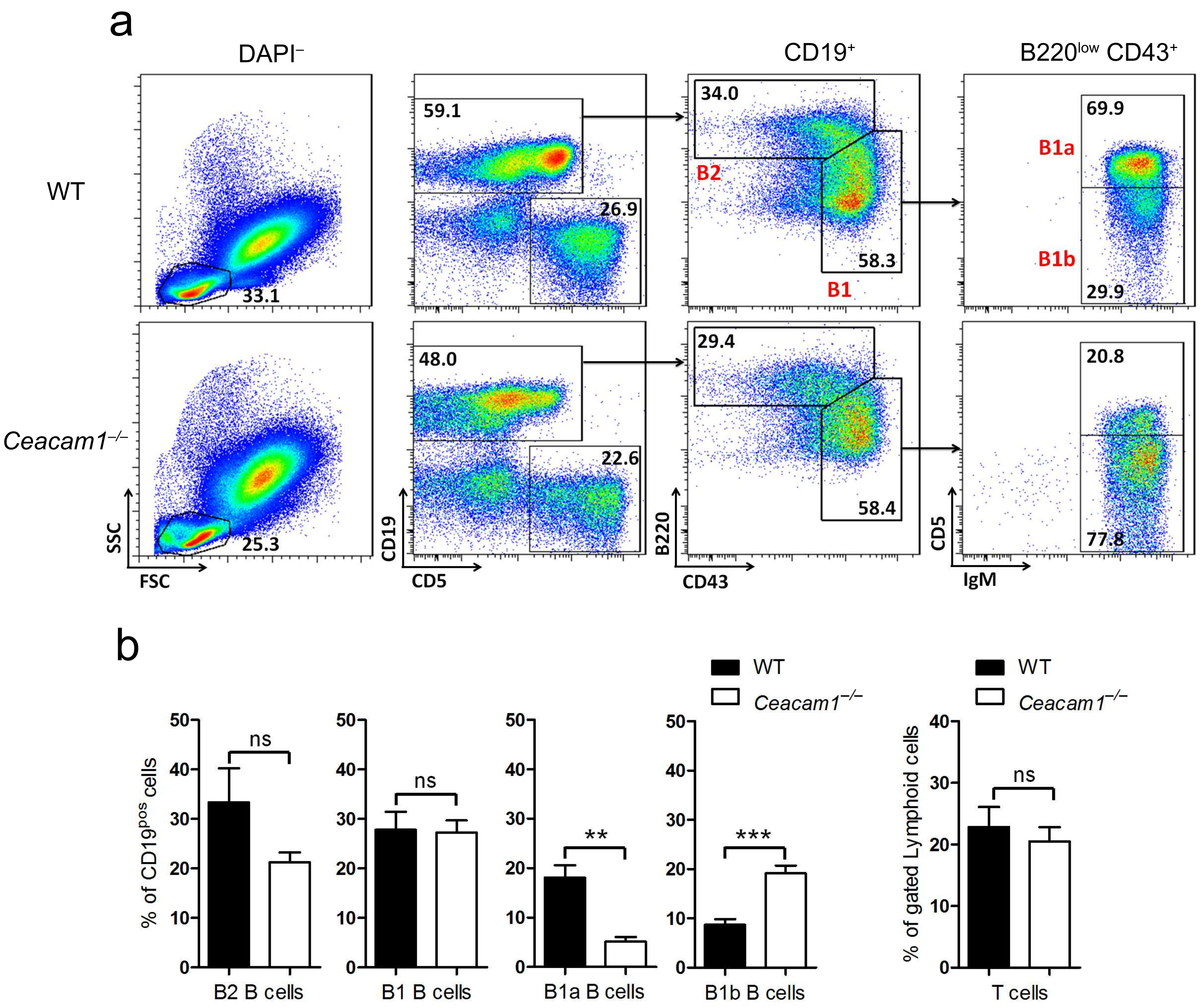

Supplementary Figure 4: CEACAM1 expression affects B1a B-cell proportion in peritoneum

a: Representative dot blots, gating strategy, and percentage of B1 B-cell subpopulations from wild-type (WT) and *Ceacam1*$^{-/-}$ mice as measured by flow cytometry in peritoneal cavity (n = 5). **b:** Bar diagram showing average frequencies of indicated subsets calculated from the gates shown above. Numbers in plots represents the percentage of events as a function of indicated parent gate. B2 ($CD19^{+}B220^{+}CD43^{-}$), B1a ($CD19^{+}B220^{+}CD43^{+}CD5^{+}$), and B1b ($CD19^{+}B220^{+}CD43^{+}CD5^{-}$). $^{**}P < 0.01$; and $^{***}P < 0.001$ (Student's t-test); ns = not significant. Data are representative of two experiments (mean ± SEM).

Supplementary Figure 5

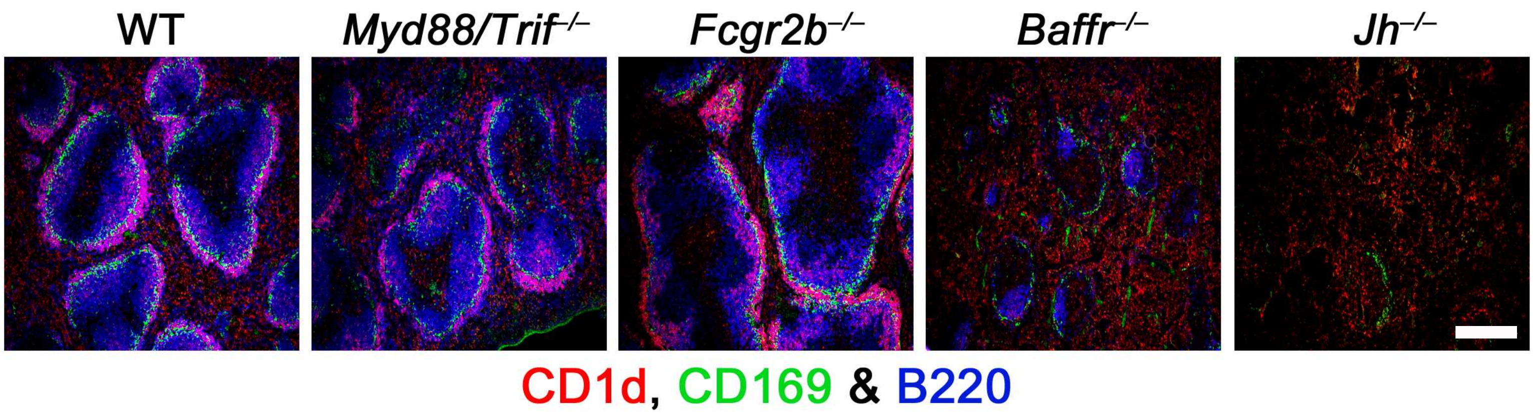

Supplementary Figure 5: BAFF receptor signaling resembles CEACAM1-mediated signaling
Representative immunofluorescence of spleen sections derived from naïve wild-type (WT) (n = 6), *Myd88/Trif*$^{-/-}$ (n = 5), *Fcgr2b*$^{-/-}$ (n = 6), *Baffr*$^{-/-}$ (n = 6), and *Jh*$^{-/-}$ (n = 6) mice after staining for marginal zone B cells (CD1d, red), marginal zone macrophages (CD169, green), and B cells (B220, blue). Scale bars, 300µm.

Supplementary Figure 6

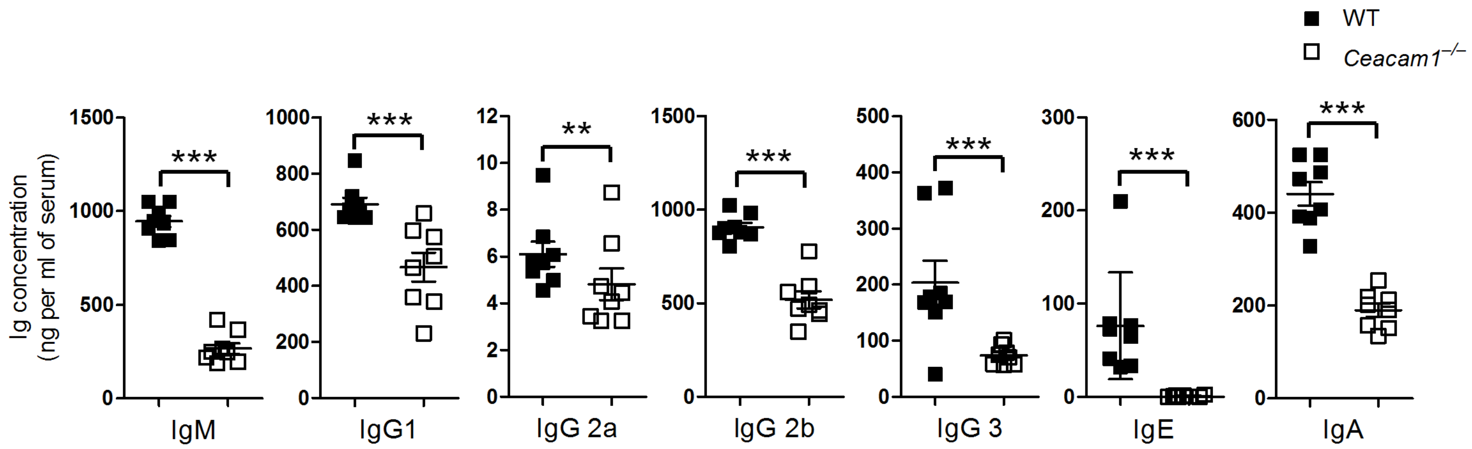

Supplementary Figure 6: CEACAM1 influences the levels of serum immunoglobulins
Histogram showing levels of various serum immunoglobulin isotypes and subtypes in naïve wild-type (WT) and *Ceacam1*$^{-/-}$ mice (n = 8 per genotype). $^{**}P < 0.01$; and $^{***}P < 0.001$ (Student's t-test). Data are representative of two experiments (mean ± SEM).

Supplementary Figure 7

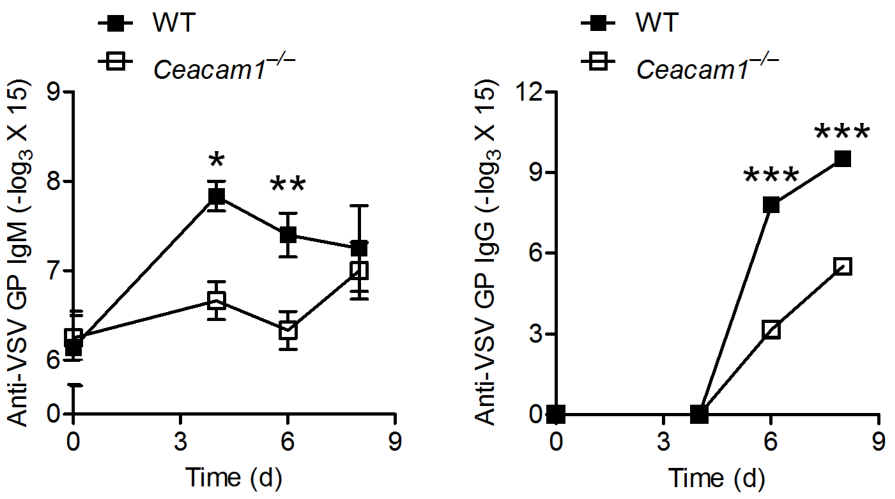

Supplementary Figure 7: CEACAM1 is essential for anti-VSV-specific Ig production Histogram showing anti-VSV–specific IgM and IgG antibodies generated in wild-type (WT) and *Ceacam1*$^{-/-}$ mice after intravenous infection with 2 × 10^6 PFU of VSV (n = 4–7 per group). **P* < 0.05; ***P* < 0.01; and ****P* < 0.001 (Student's t-test). Data are representative of two experiments (mean ± SEM).

Supplementary Figure 8

a

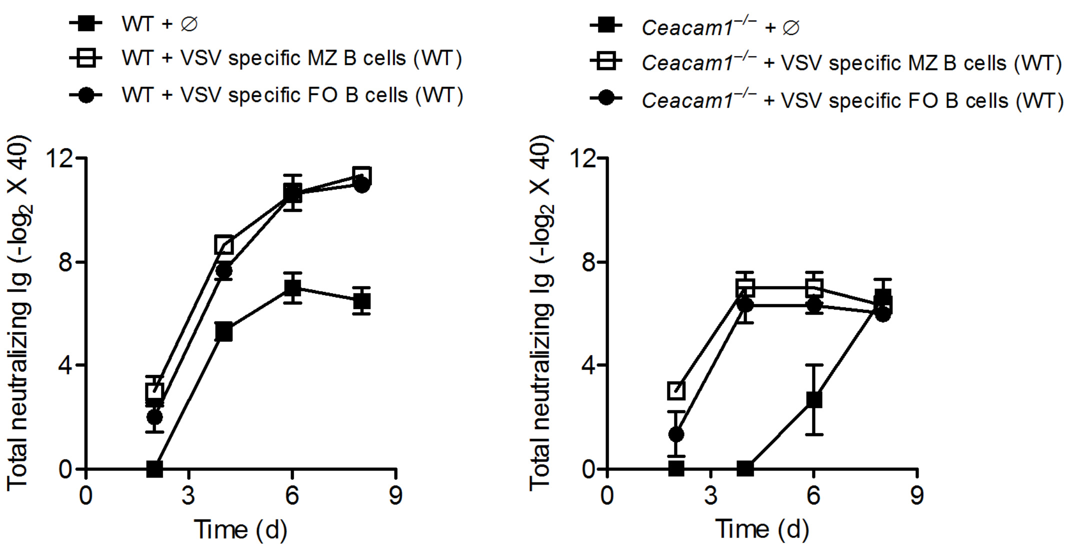

b

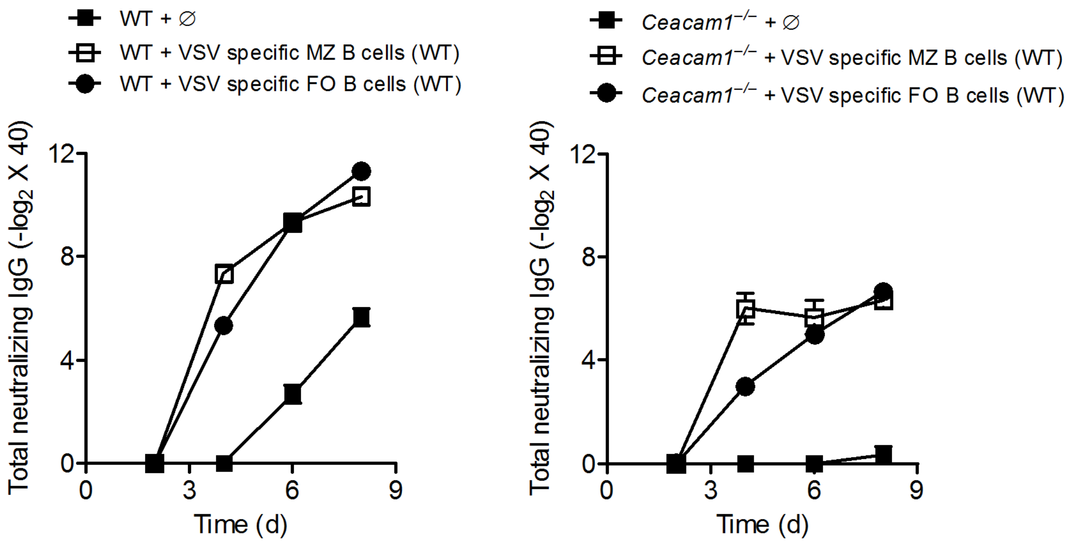

Supplementary Figure 8: MZ and FO B cells can rescue survival in *Ceacam1*$^{-/-}$ mice
Histogram showing total VSV neutralizing antibody response (**a**) and neutralizing IgG antibodies (**b**) measured in sera of wild-type (WT) and *Ceacam1*$^{-/-}$ mice that also received 10 × 10^6 MZ and 1 × 10^6 FO VSV-specific B cells (Vi10) on day -1 and were then intravenously infected with 2 × 10^6 PFU VSV on day 0 (n = 3 per group).

Supplementary Figure 9

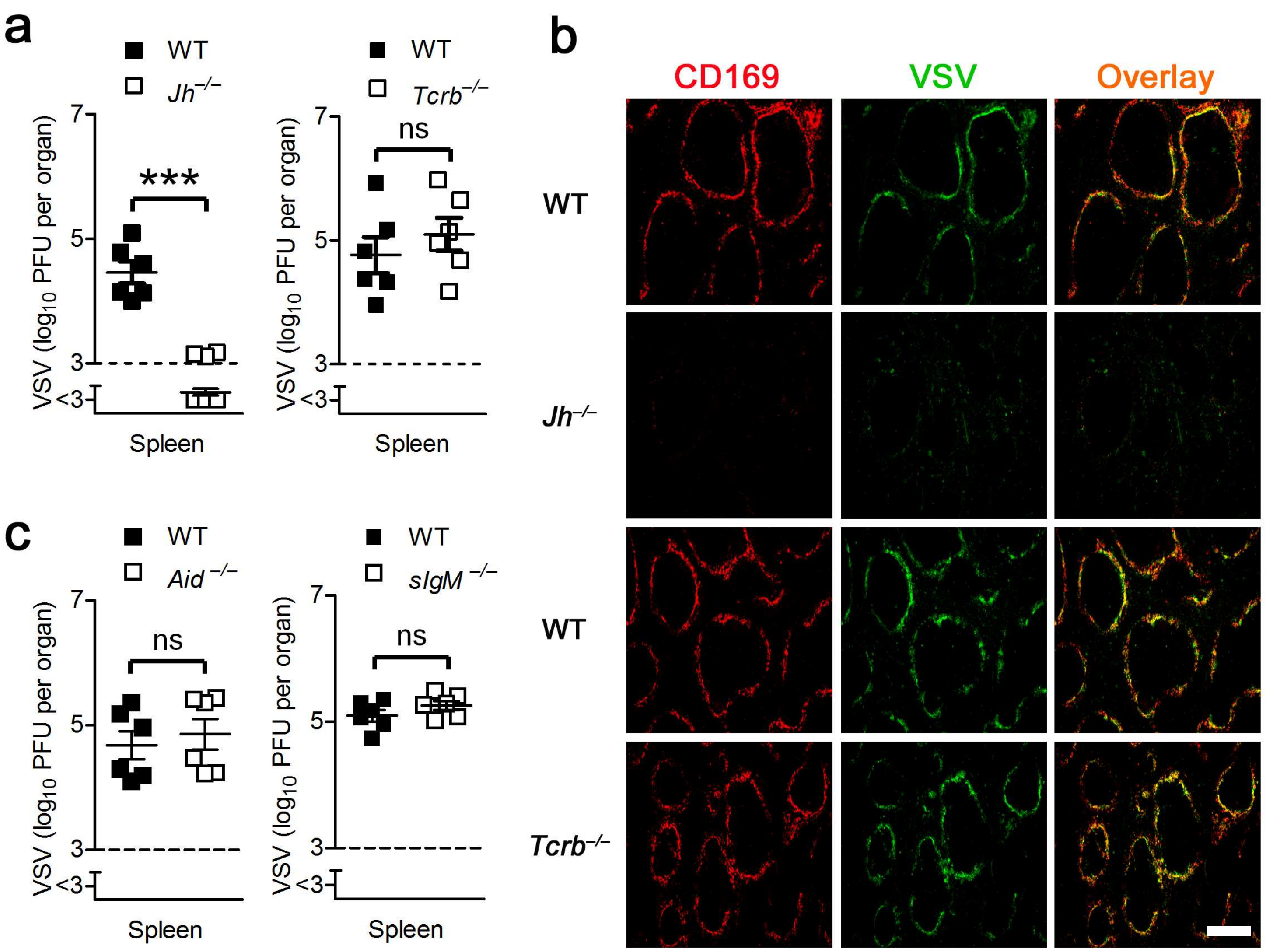

Supplementary Figure 9: B cells but not other cell types are important for the replication of vesicular stomatitis virus in the spleen and the activation of adaptive immunity. a: Spleen vesicular stomatitis virus (VSV) titers from wild-type (WT) or *Jh*$^{-/-}$ mice or from WT or *Tcrb*$^{-/-}$ mice (n = 6 per genotype) 7 h after intravenous infection with 2×10^6 PFU of VSV. **b:** Immunofluorescence assay of spleen sections from WT or *Jh*$^{-/-}$ mice or from WT or *Tcrb*$^{-/-}$ mice 7 h after intravenous infection with 2 x 10^8 PFU of VSV stained with VSV glycoprotein (green) and CD169 (red). One of six representative slides is shown. **c:** Spleen VSV titers from WT or *Aid*$^{-/-}$ mice or from WT or *sIgM*$^{-/-}$ mice (n = 6 per genotype) 7 h after intravenous infection with 2×10^6 PFU of VSV. Scale bars, 300 µm. ****P* < 0.001 (Student's t-test); ns = not significant. Data are representative of two (a & c) experiments. One representative slide of six (b) slides is shown (mean ± SEM, a, c).

Supplementary Figure 10

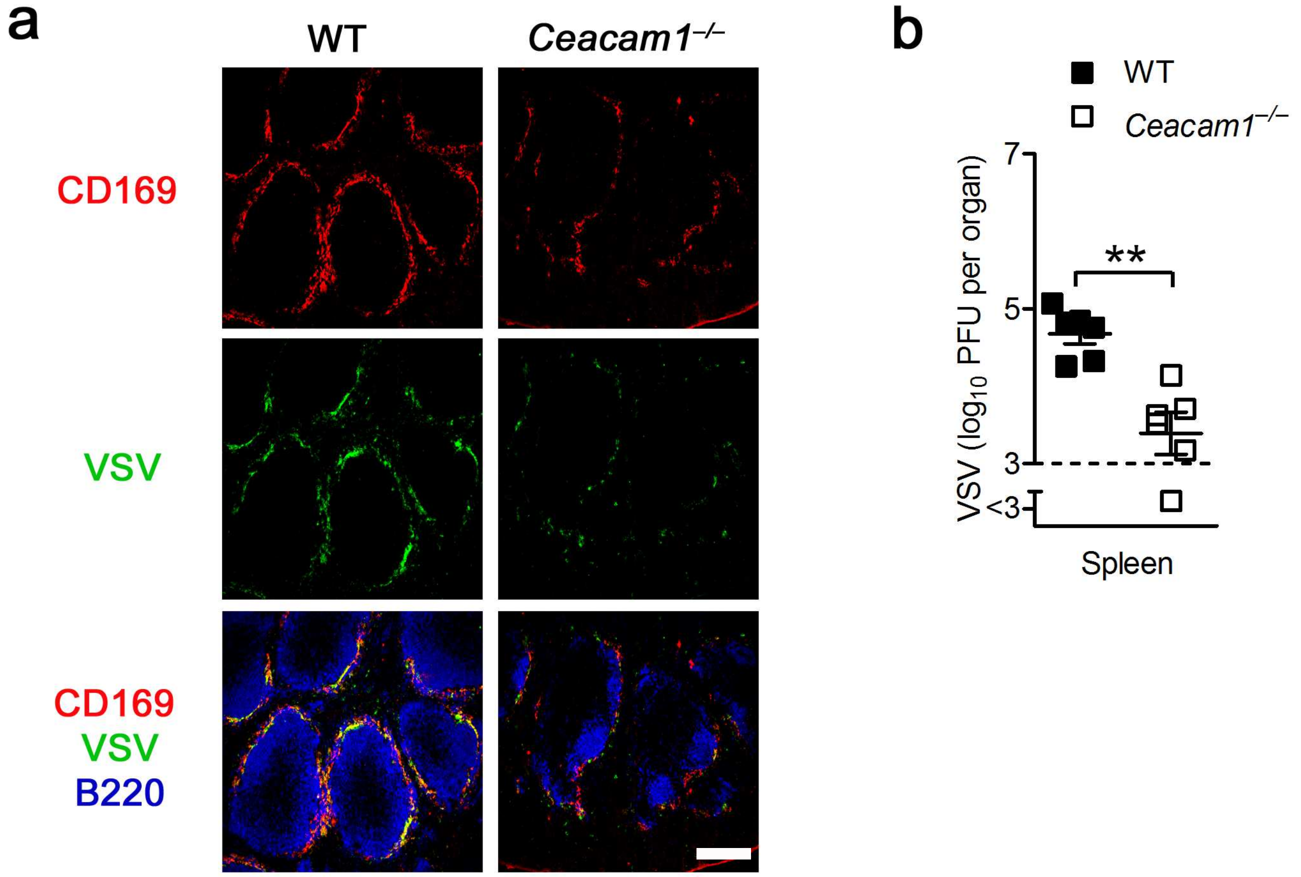

Supplementary Figure 10: Deficient marginal zone in *Ceacam1*$^{-/-}$ mice limits antiviral innate immune response

a: Immunofluorescence of spleen sections from wild-type (WT) or *Ceacam1*$^{-/-}$ mice 7 h after intravenous infection with 2 × 10^8 PFU of vesicular stomatitis virus (VSV), stained for VSV glycoprotein (green), CD169 (red), and B220 (blue) (n = 6 per genotype). One of six representative slides is shown. Scale bar, 300 µm. **b:** Spleen VSV titers from WT or *Ceacam1*$^{-/-}$ mice 7 h after intravenous infection with 2 × 10^6 PFU of VSV (n = 6 per genotype). ***P* < 0.01 (Student's t-test). Data are representative of two (b), or one of two (a) experiments (mean ± SEM, b).
